# Cancer in transition: discovery of tumor-intrinsic transcriptional programs shaping the immune and microenvironmental landscape

**DOI:** 10.1186/s40364-026-00925-z

**Published:** 2026-04-16

**Authors:** Alf Spitschak, Rosaely Casalegno Garduño, Brigitte M. Pützer

**Affiliations:** 1 Institute of Experimental Gene Therapy and Cancer Research, Rostock University Medical Center, 18057 Rostock, Germany; 2 Department Life, Light & Matter, University of Rostock, 18059 Rostock, Germany

**Keywords:** Cancer biomarkers, Transcriptional regulation, Epigenetic reprogramming, E2F1, Noncoding RNA, Tumor microenvironment, Immunotherapy biomarkers, Tumor plasticity, Multi-omics integration, Artificial intelligence in oncology

## Abstract

Cancer biomarker discovery has traditionally focused on individual molecular features; however, tumor behavior and therapeutic response are governed by integrated transcriptional and epigenetic programs that shape immune and microenvironmental states. Deciphering the determinants of metastatic evolution and the complexity of cancer ecosystems is imperative for designing novel preventive and targeted therapies. Tumor complexity, driven by intrinsic cellular heterogeneity and the dynamic plasticity of cancer cells in response to microenvironmental cues, complicates therapeutic strategies based solely on defined molecular or genetic traits. Consequently, there is an urgent need for reliable predictive biomarkers that reflect cancer vulnerabilities, indicate disease progression, or predict patient-specific therapeutic responses to enable truly individualized treatment strategies. Current biomarkers encompass genetic and epigenetic alterations, non-coding RNAs, epithelial-mesenchymal transition-, stemness-, and metastasis-associated transcription factors, as well as cellular components of the tumor microenvironment, including immune cell subsets and cancer-associated fibroblasts. In immuno-oncology, additional biomarkers such as tumor mutational burden, mismatch repair deficiency/microsatellite instability-high status, and PD-L1 expression are widely used for patient stratification. Importantly, the reversible nature of epigenetic modifications, aberrant transcription factor activity, and cell-intrinsic signaling alterations, together with dynamic interactions within the tumor microenvironment, profoundly influence cancer behavior and treatment outcomes. This review summarizes recent advances in cancer and immune-related biomarker research. It outlines a regulatory, systems-level framework that integrates tumor-intrinsic gene control programs with multi-omic, cellular, spatial, and AI-enabled biomarkers. This framework aims to capture tumor plasticity more effectively and advance precision oncology.

## Background

Cancer is a multifactorial disease driven by genetic mutations, epigenetic modifications, and dysregulated signaling pathways. It is one of the leading causes of morbidity and mortality worldwide. According to the World Health Organization (WHO), cancer is responsible for approximately 10 million deaths per year, accounting for about one-sixth of all deaths globally. As populations age and life expectancy increases, the likelihood of a significant rise in cancer prevalence grows. Consequently, the identification and validation of solid indicators of cancer in the body is crucial for the development of targeted therapies, early detection and diagnosis, patient stratification for treatment, outcome prediction, and assessment of therapy efficacy. Fortunately, malignant tumors can be detected through specific traits produced by cancer cells (e.g., exosomes, lncRNASs), cancer stem/progenitor cells (CSCs), and circulating tumor cells (CTCs), or by alterations in surrounding microenvironmental cells responding to the tumor, such as cytotoxic T lymphocytes (CTLs), tumor-associated macrophages (TAMs), and cancer-associated fibroblasts (CAFs). These so-called cancer biomarkers are biological molecules found in tissues and/or body fluids that indicate abnormal processes linked with progression, metastases, and therapy resistance. Testing can be performed using a single biomarker via fluorescence in situ hybridization (FISH), immunohistochemistry (IHC), Sanger sequencing or PCR. However, because genetic material from tumors can be limited, the most efficient use of samples can be achieved through multiple-biomarker testing, such as next-generation sequencing (NGS), where different biomarkers are tested simultaneously to obtain more comprehensive data and facilitate more informed therapeutic decisions.

Currently, cancer detection in most parts of the world relies primarily on the onset of patient-reported symptoms, which are often non-specific and overlap with benign conditions, making early diagnosis particularly challenging. As a result, definitive diagnosis typically occurs only after follow-up examinations such as biopsies, CT scans, or X-rays are performed. Many cancers remain asymptomatic and undetected during their early stages in the absence of clear clinical symptoms, contributing to delayed diagnosis and reduced treatment effectiveness. Ovarian cancer, for instance, has a poor prognosis, mainly due to its late detection, which results in a five-year survival rate of 20% in advanced stages [1] , and is often misdiagnosed [2]. Similarly, pancreatic cancer is rarely detected before reaching stage IV, resulting in an extremely low five-year survival rate of only 7%, one of the lowest among all aggressive cancers [3]. This underscores the urgent need for more sensitive and specific diagnostic tools capable of identifying malignancies before obvious symptoms appear. Here, new artificial intelligence (AI) models that integrate biosensors for detecting specific biomarkers in cancer cells can be a valuable diagnostic tool [4]. Standardized biomarkers that can be detected in the early stages of cancer are the focus of intense research. The identification of cancer biomarkers has advanced significantly with the recognition of distinct genetic signatures resulting from alterations in cancer-associated genes, such as translocations, amplifications, and mutations. Currently, biomarkers encompass various molecular entities, including DNA, RNA, small molecules, proteins/peptides, hormones, transcription factors, and metabolites [5]. Comparative transcriptomic analyses between tumor samples and paired healthy tissue have revealed differentially expressed genes (DEGs) implicated in key biological processes such as DNA replication and cell cycle regulation as well as cellular components like the mitotic spindle and microtubule cytoskeleton [6]. Aberrant overexpression of several genes that contribute to metastasis, including *E2F1*, *ADAM9*, *MTA1*, *SP1*, *KLF5*, *BIRC5*, *CD44*, *ATF4*, *SERPINE2*, and *SLPI*, has been observed in multiple cancer entities [7, 8]. Cancer-driver genes have been further identified through genomic sequencing approaches, particularly NGS, RNA sequencing (RNA-seq), whole-genome sequencing (WGS), and whole-exome sequencing (WES). NGS allows high-throughput sequencing of large amounts of DNA or RNA and is primarily used in patients with metastatic disease [9]. The protein-coding regions (exons) of the genome are sequenced by WES to detect mutations that directly affect protein structure and function [10, 11], while WGS provides a comprehensive view of the genome, encompassing both coding and non-coding regions [5]. Various techniques are employed to detect overexpression of proteins in cancer cells, including IHC, multiplex IHC, multiplex immunofluorescence (mIF), enzyme-linked immunosorbent assay (ELISA), and flow cytometry [5]. In clinical practice, a wide range of predictive and prognostic biomarkers for various solid tumors are routinely examined by IHC. Examples include programmed death-ligand 1 (PD-L1), human epidermal growth factor receptor 2 (HER2), the proliferation marker Ki-67, anaplastic lymphoma kinase (ALK), ROS1, DNA mismatch repair (MMR) proteins, neurotrophic tyrosine receptor kinase (NTRK), progesterone receptor (PR), and estrogen receptor (ER). ER was identified as the first predictive biomarker and is still the most commonly used biomarker to guide endocrine therapy decisions. ELISA is often used to detect tumor-associated antigens (TAAs) such as carcinoembryonic antigen (CEA) and prostate-specific antigen (PSA) in patients with colorectal and prostate cancer, respectively. Each of these markers contributes to patient stratification, therapeutic decision-making and cancer prediction [5].

The precision of cancer biomarkers is largely determined by their specificity, i.e. their ability to clearly distinguish cancer cells from healthy tissue [12]. However, accurately predicting which patients will respond to targeted therapies remains a significant clinical challenge [13]. The lack of specificity of cancer-derived biomarkers remains a major limitation. Moreover, misinterpretation of such indicators of malignancy can lead to incorrect patient treatment [5]. To overcome these limitations, multiparametric measurements should be considered that combine both tumor-immune features/interactions as predictive markers and targets for successful therapy [14]. The accuracy of biomarkers in precision oncology will ultimately be reflected in cost-effective treatment and minimal toxicity. Ultimately, biomarkers serve not only as disease identifiers, but also as therapeutic endpoints to specifically block tumor growth or metastasis. Based on the identified biomarker, tailored therapeutic strategies can be developed to combat the molecular characteristics of cancer cells, such as gene mutations, protein overexpression, or disruptions in key signaling pathways. This approach enables precision medicine, in which treatments are specifically designed to interfere with cancer-driving mechanisms. This improves therapy effectiveness, minimizes off-target effects, and optimizes clinical outcomes for individual patients. Also, biomarker testing is a dynamic process that allows for adaptation to changes in cancer. For example, tyrosine kinase inhibitors are used to treat lung carcinoma with epidermal growth factor receptor (EGFR) mutations. However, if resistance develops ctDNA testing can reveal the presence of new mutations so that treatment can be adjusted accordingly [15].

In this review, we integrate recent advances in diagnostic, prognostic, and predictive cancer biomarkers from a regulatory and systems-level perspective. Rather than treating biomarkers as isolated molecular entities, we focus on how tumor-intrinsic transcriptional and epigenetic programs give rise to coordinated changes in gene expression, immune composition, stromal remodeling, and spatial organization within the tumor microenvironment (TME). We discuss molecular signatures linked to epithelial-mesenchymal transition (EMT), stemness, immune evasion, and therapy resistance as downstream manifestations of these regulatory networks, encompassing transcription factors, non-coding RNAs, epigenetic modifiers, and cellular TME components such as immune cells and cancer-associated fibroblasts. Finally, we examine current limitations in biomarker implementation and highlight emerging experimental and computational strategies, including multi-omics integration and artificial intelligence, to improve patient stratification, therapeutic decision-making, and precision oncology outcomes.

## Genetic and epigenetic drivers of cancer: diagnostic, therapeutic and biomarker implications

**Fig. 1:**
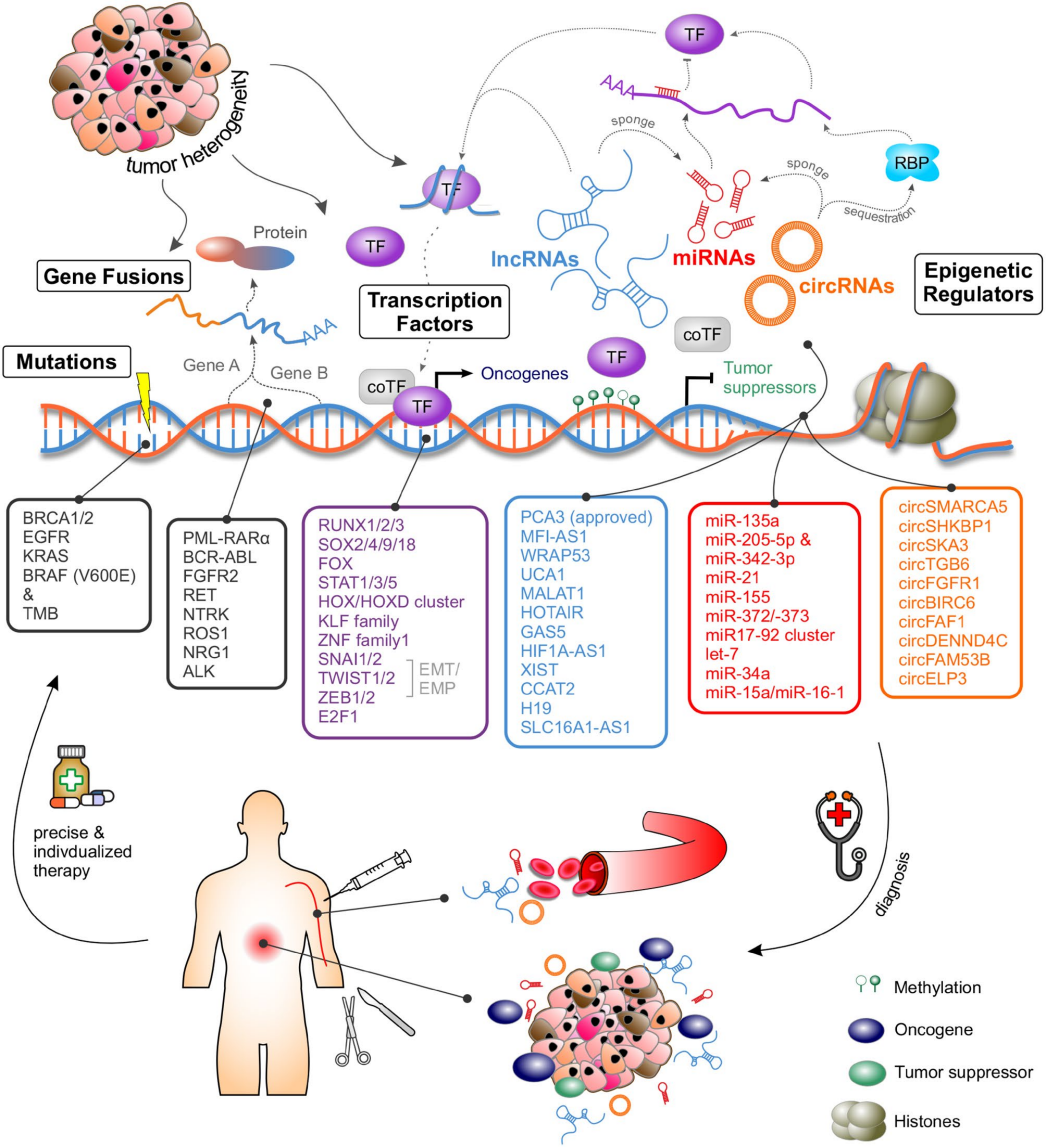
Genetic and epigenetic regulators as biomarkers for diagnosis and therapy in cancer

Cancer develops because of cumulative genetic mutations that impair the activity of oncogenes and tumor suppressor genes (TSGs), which either promote or inhibit cell division. These permanent alterations in the DNA sequence of a gene are also closely related to epigenetic changes [16, 17]. In fact, the combination of genetic and epigenetic characteristics of cancer is therapeutically relevant [17, 18]. Genomic profiling enables the identification of specific mutations that drive cancer progression by altering protein activity through either overactivation or loss of function, thereby maintaining the survival of malignant cells. Consequently, targeted therapies must be tailored to these specific genetic alterations. However, beyond gene mutations, the epigenetic landscape plays a pivotal role in promoting uncontrolled cell proliferation and metastasis. These epigenetic modifications not only serve as potential therapeutic targets but also hold promise as clinically relevant biomarkers for cancer detection and stratification [19]. Molecular-targeted therapies aim to selectively disrupt these oncogenic changes, enabling precise intervention at the level of the genetic drivers that sustain tumor progression (Fig. 1).

### Genetic mutations

Gene mutations play a crucial role in cancer progression and can serve as robust predictive biomarkers and clinically relevant targets [15]. They can be either germline or somatic in origin. Germline mutations are inherited and present in every cell of the body. Testing for germline mutations typically involves analysis of DNA extracted from blood or saliva samples. In contrast, somatic mutations are acquired alterations that occur during a person’s lifetime and can lead to cancer development. These are identified through the genomic testing of biopsies or tumor tissue.

Approximately 20% of all ovarian cancer cases are associated with inherited mutations in cancer susceptibility genes, most notably *BRCA1* and *BRCA2* [20]. These genes encode DNA repair proteins involved in the homologous recombination (HR) repair pathway, and their inactivation causes genomic instability and tumor development [21]. Importantly, tumors harboring *BRCA1/2* mutations exhibit increased sensitivity to DNA-damaging agents such as platinum-based chemotherapies. This vulnerability has led to the development of therapies targeting DNA repair pathways, particularly poly (ADP-ribose) polymerase (PARP) inhibitors [22–24]. PARP inhibitors exploit the concept of synthetic lethality and selectively induce cell death in cancer cells lacking HR repair mechanisms. In a pivotal clinical trial, ovarian cancer patients with *BRCA1/2* mutations who had responded completely or partially to platinum-based chemotherapy and subsequently received PARP inhibitors experienced a 70% reduction in the risk of disease progression or death [25]. Furthermore, tumors with homologous recombination deficiency (HRD), even in the absence of *BRCA* mutations, also show increased sensitivity to PARP inhibition, broadening the clinical applicability of this therapeutic approach.

Prophylactic measures play a major role in cancer prevention in hereditary cancer syndromes, especially those involving BRCA1/2 mutations [26]. If a patient is diagnosed with ovarian cancer and found to carry *BRCA* mutations, her first-degree relatives have a 50% probability of carrying the same mutation due to autosomal dominant inheritance. Therefore, these relatives are strongly advised to undergo genetic testing to determine their mutation status and associated cancer risk. Furthermore, carriers of *BRCA1/2* mutations have a markedly increased cumulative lifetime risk of developing breast and/or ovarian cancer, with the risk increasing significantly with age [27]. Prophylactic strategies are recommended for individuals who have tested positive for a *BRCA* mutation but have not yet developed cancer. Depending on the individual’s risk profile and clinical context, these include enhanced screening or risk-reducing surgery, such as prophylactic mastectomy or salpingo-oophorectomy [26]. Genetic testing for hereditary cancer risk is increasingly being performed using multi-gene panel testing, which allows the simultaneous analysis of several genes associated with cancer predisposition [20, 28]. This approach improves the detection of at-risk individuals beyond *BRCA1/2* and facilitates more comprehensive risk assessment and personalized preventive care.

In addition to *BRCA1/2*, numerous somatic mutations serve as predictive biomarkers that guide the selection of targeted therapies for various types of cancer. Comprehensive reviews and curated databases summarize the rapidly expanding landscape of actionable genomic alterations in precision oncology [29, 30]. A well-known example is the *EGFR* mutation, which plays a critical role in NSCLC pathogenesis [31, 32]. *EGFR* mutations, particularly in exons 19 and 21, are associated with increased sensitivity to EGFR tyrosine kinase inhibitors (TKIs) such as osimertinib, gefitinib, and erlotinib. The presence of these mutations serves as a biomarker for identifying patients who are likely to benefit from EGFR-targeted therapies [32–34]. In colorectal cancer, the wild-type exon 2 of the Kirsten rat sarcoma virus oncogene homolog *(KRAS)* is a decisive determinant for the use of monoclonal anti-EGFR antibodies such as cetuximab and panitumumab. KRAS mutations downstream of the EGFR pathway render these therapies ineffective. Therefore, the absence of KRAS mutations is a prerequisite for cetuximab administration in metastatic colorectal cancer [35]. Another key example is BRAF mutations, which are found in approximately 50% of melanomas [36]. This mutation leads to the constitutive activation of the MAPK/ERK signaling pathway and promotes uncontrolled cell proliferation. Vemurafenib, a selective *BRAF*^*V600*^ inhibitor, was developed to specifically target tumors with this mutation, resulting in a significant improvement in progression-free and overall survival (OS) in melanoma patients. Unfortunately, most patients eventually develop therapeutic resistance, highlighting the need for alternative treatment strategies [37].

These representative examples illustrate the clinical relevance of genetic profiling in oncology treatment, where the identification of specific driver mutations facilitates tailored therapies based on precise molecular characteristics of the tumor.

### Gene fusions and amplifications

Gene fusions are particularly valuable diagnostic biomarkers in oncology that serve as molecular signature identifiers for specific cancer subtypes. Their identification helps classify the disease and guide therapeutic decisions. For instance, a subgroup of patients with acute myeloid leukemia (AML) identified by PML-RAR fusions shows exceptional sensitivity to retinoic acid and arsenic. Similarly, the long-known Philadelphia chromosome, which is present in 95% of patients with chronic myeloid leukemia (CML), can be used as a guide for treatment decisions. This abnormality arises from the translocation of *Abl* from chromosome 9 to chromosome 22, creating an abnormal *BCR-Abl* gene that encodes a highly active tyrosine kinase protein increasing cell proliferation, differentiation, and survival. Thereafter, BCR-Abl tyrosine kinase inhibitors are used as effective treatment with minimal side effects and higher survival rates for over 80% of patients [15].

Recently, additional gene fusions have been identified as predictive biomarkers for targeted therapies (Fig. 1). Notable examples include fibroblast growth factor receptor 2 (*FGFR2*), rearranged during transfection (*RET*) proto-oncogene, neurotrophic receptor tyrosine kinase 3 (*NTRK3*), the proto-oncogene tyrosine protein kinase (*ROS1*), the neuregulin-1 gene (*NRG1*), and anaplastic lymphoma kinase (*ALK*) fusions [5, 38–43]. Many of these rearrangements function as oncogenic drivers that constitutively activate receptor tyrosine kinase signaling pathways and thereby define tumors that are highly sensitive to targeted inhibition. ALK or ROS1 fusions, particularly in NSCLC, predict strong responses to selective tyrosine kinase inhibitors such as crizotinib or alectinib. Likewise, NTRK fusions, although rare, make tumors remarkably sensitive to highly selective TRK inhibitors such as larotrectinib, which has demonstrated efficacy across multiple tumor types in tumor-agnostic clinical trials. These alterations can occur across diverse cancers, so their detection relies on comprehensive next-generation sequencing approaches that enable fusion detection and facilitate precision oncology strategies [44–47].

Gene amplification represents another important class of predictive biomarkers in cancer. For example, MYCN amplification is associated with aggressive disease in neuroblastoma, while MET amplification can act as an oncogenic driver and a mechanism of resistance to EGFR-targeted therapies in lung cancer [48, 49].

Another well-established example is HER2 amplification, which is often detected by FISH. HER2 is overexpressed in multiple malignant tumors, including breast, ovarian, prostate, gastric, colorectal, and lung cancers, where it promotes aggressive tumor growth. Targeted inhibition of HER2 signaling with trastuzumab in HER2-positive breast cancer has significantly improved clinical outcomes while minimizing chemotherapy-associated toxicity, exemplifying the effectiveness of personalized therapies in precision oncology [15, 50–52].

### Epigenetic biomarkers

Epigenetic mechanisms are crucial for gene expression regulation and sustaining genomic stability, enabling cells to adapt to a changing environment. These mechanisms, including DNA and RNA methylation, histone modifications, chromatin remodeling, and non-coding RNAs (ncRNAs), are frequently dysregulated in cancer development and can be heritable (Fig. 1). A significant majority, approximately 90–95%, of pathological conditions exhibit epigenetic alterations, underscoring the critical role of epigenetic regulation in disease etiology [53]. Consequently, they have emerged as a central focus for understanding the intrinsic landscape behind tumor initiation, progression, and resistance to therapy, and are key determinants for cancer diagnosis, prognosis, and therapeutic decision-making. Since these modifications do not alter the DNA sequence, they are potentially reversible, making them attractive targets for precision oncology [16, 17, 19, 54]. Imbalances in histone methylation and demethylation, for example, are involved in cancer progression. Furthermore, reduced acetylation of histones and other proteins, a key feature of epigenetic silencing, may exacerbate cancer malignancy [19].

In addition, DNA methylation, a chemical modification of DNA in which methyl groups are attached to cytosine or adenine, can silence tumor suppressor genes or activate oncogenes [55]. This occurs predominantly in CpG islands and can potentially be used to determine the cancer stage. Gene expression is partially regulated by DNA methylation, whereby silencing usually occurs as a result of the addition of methyl groups that prevent the binding of transcription factors (TFs) to DNA sites [16, 19]. In this context, hypermethylation has been associated with higher rates of prostate cancer. Moreover, hypermethylated CpG regions have a higher probability of mutation and are involved in different cancers. Following gene expression profiling and DNA methylation analysis of periprostatic adipose tissue from patients with prostate cancer, a gene signature of 30 genes was identified that can be used to determine whether the cancer is primary or advanced. This signature has been validated in published cohorts, demonstrating its potential as a prognostic biomarker for estimating the risk of progression and metastasis. More specifically, six genes (namely *POLR3K, EEF1D, IGFALS, H2AW, WRAP73* and *FASTK)* demonstrated a pronounced mutation burden in the two stages [56]. On the other hand, hypomethylated oncogenes have been linked to cancer development, such as glioblastoma, papillary thyroid carcinoma, and colorectal cancer. Moreover, the deregulation of cancer-related genes in the early preneoplastic phase involves both hyper- and hypomethylation processes in HCC [57].

### Long non-coding RNAs

Non-coding RNAs represent more than 70% of the human genome and are imperative epigenetic regulators of gene expression. They include long noncoding RNAs (lncRNAs), microRNAs (miRNAs), and circular RNAs (circRNAs), which have multifaceted roles in promoting tumor proliferation, invasion, metastasis, angiogenesis, and immune evasion [58]. Recent advances in exploring their function in cancer biology have demonstrated their potential as biomarkers and therapeutic targets [59]. NcRNAs are stable, abundant, and conserved molecules that are often expressed in a tissue/developmental stage-specific manner and can be detected in body fluids. Each of them has a unique mechanism of action [60].

LncRNAs function as gene expression regulators, frequently interacting with epigenetic factors to shape the outcome of many biological processes including cancer. Over the last few years, growing evidence has supported them as valuable markers for the clinic [61] (Table 1, Fig. 1). Prostate cancer antigen 3 (PCA3) is a prominent example of a clinical success story in prostate cancer diagnosis. Its detection in the urine of prostate cancer patients correlates with disease severity [62–64]. PCA3 is FDA-approved and routinely used in clinical practice [65–67]. Several other lncRNAs are being evaluated as diagnostic biomarkers, such as MFI2–AS1 in kidney cancer (NCT04946266), and WRAP53 together with UCA1 in hepatocellular carcinoma (NCT05088811) [61, 68, 69]. Another well-characterized example is metastasis-associated lung adenocarcinoma transcript 1 (MALAT1), which is frequently overexpressed in multiple malignancies, including lung, breast, colorectal, gastric, thyroid, and hematological cancers, where it regulates a broad range of cancer- and metastasis-related processes like proliferation, invasion, EMT, angiogenesis, and drug resistance through various signaling pathways and by sponging miRNAs [70–80]. It´s key role in colorectal cancer metastasis, for instance, is based on promoting EMT and regulating alternative splicing events that facilitate cancer progression. MALAT1 can be detected in tumor tissue, serum, plasma, and exosomes, supporting its use as a non-invasive biomarker [71, 80, 81]. The detection of MALAT1 levels from peripheral blood cells could reflect the presence of NSCLC with a specificity of 96% [82]. Moreover, MALAT1 levels provide prognostic value for overall and disease-free survival [83, 84]. Expression of the oncogenic HOX transcript antisense intergenic RNA (HOTAIR) is elevated in glioblastoma, breast, and colorectal cancer (CRC), and correlates with advanced tumor stage, lymph node metastasis, recurrence, and poor survival outcomes [85–91]. The ncRNA promotes tumorigenesis by regulating gene expression through epigenetic mechanisms e.g. by directly interacting with EZH2 or acts as a competitive endogenous RNA (ceRNA) sponge and also modulates Wnt/β-catenin, PI3K/Akt/mTOR, and EMT-related cascades [91]. Changes in circulating HOTAIR levels reflect treatment response and disease progression underscoring its utility in monitoring chemotherapy efficacy and relapse [87, 89, 92–95]. Besides, other lncRNAs have been identified as stable blood markers for NSCLC diagnosis (GAS5, HIF1A-AS1, and XIST), poor prognosis biomarkers (CCAT2, CARLo-5), and molecular therapy targets such as H19 (reviewed by [96]).

**Table 1:** Overview of cancer-related non-coding RNAs investigated in clinical trials

ncRNA	class	Drug name	Cancer type	Identifier
PCA3	lncRNA		Prostate cancer	Approved
MFI2–AS1	lncRNA		Kidney Cancer	NCT04946266 (observational)
WRAP53 & UCA-1	lncRNA		Hepatocellular Carcinoma	NCT05088811 (observational)
H19	lncRNA	BC-819 (DTA-H19, H19-DTA)	Pancreatic, Bladder & Ovarian Cancer, Hepatocellular Carcinoma	NCT00711997 (Phase1/2) NCT01413087 (Phase 2) NCT00393809 (Phase 1/2) NCT00595088 (Phase 2) NCT00826150 (Phase 1/2) NCT04767750 (observational)
miR-34a	miRNA	MRX34	Liver cancer, lymphoma, melanoma, renal cell carcinoma, NSCLC, SCLC	NCT01829971 (Phase 1), NCT02862145 (Phase 1/2)
miR-16	miRNA	TargomiR (MesomiR1)	MPM, NSCLC	NCT02369198 (Phase 1)
miR-155	miRNA	Cobomarsen (MRG-106)	CTCL, CLL, DLBCL, ATLL	NCT02580552 (Phase 1), NCT03713320 (Phase 2)
miR-193a-3p	miRNA	INT-1B3	TNBC, NSCLC, melanoma, colon cancer, HCC	NCT04675996 (Phase 1)
miR-10b	miRNA	TTX-MC138	Advanced solid tumors	NCT05908773 (early Phase 1), NCT06260774 (Phase 1/2)
circFAF1 (hsa_circ_100219); circELP3 (hsa_circ_0001785)	circRNA		Breast Cancer	NCT05771337 (observational)
circFAM53B	circRNA	CircFam53B-219aa DC vaccine (camrelizumab)	Breast neoplasms Solid Tumors	NCT06530082 (Phase 1), NCT07245901 (Phase 1/2)
circDENND4C	circRNA		Ovarian Cancer	NCT06617585 (observational)
hsa_circ_0004001	circRNA		Hepatic Cell Carcinoma	NCT06042842 (observational)

We combined high-throughput transcriptomic analyses with bioinformatics and structure modeling to search for lncRNAs involved in E2F1-mediated prometastatic GRNs. We found that lncRNA SLC16A1–AS1, as a target and coactivator of this transcription factor, promotes metabolic plasticity and reprogramming of bladder cancer cells to drive cancer progression to invasive stages [97]. Mechanistically, SLC16A1–AS1, which is transactivated by E2F1 together with its associated lactate transporter-encoding gene SLC16A1/MTC1, forms an lncRNA-protein complex with E2F1 that enhances metabolic gene expression, including SLC16A1/MTC and PPARA [97]. Moreover, other candidates in this class of ncRNAs function as TSGs, such as tumor suppressor candidate 7 (TUSC7) and LOC285194, which are associated with unfavorable disease prognosis when they are weakly expressed in lung cancer tissue [98].

### MicroRNAs

MicroRNAs (miRNAs) are 18–25 nucleotide ncRNAs that regulate gene expression post-transcriptionally and are broadly implicated in cancer initiation, progression, and metastasis [99]. Their dysregulation results from chromosomal alterations such as deletions, amplifications, or translocations, as well as mutations or single nucleotide polymorphisms (SNPs) affecting miRNA sequences or their target sites. Epigenetic mechanisms, including DNA methylation and histone modifications, can silence tumor-suppressive miRNAs or activate oncogenic miRNAs (onco-miRs) [100], while defects in miRNA biogenesis further contribute to aberrant expression [101]. These alterations generate cancer-specific miRNA profiles that serve as promising diagnostic, prognostic, and predictive biomarkers [99].

Depending on the cellular context, miRNAs can function as TSGs or oncogenes. For instance, overexpression of miR-135a is associated with poor OS in gastric cancer by inhibiting E2F1-induced apoptosis and the Sp1/DAPK2 pathway [102], and miR-205-5p together with miR-342-3p suppresses E2F1-mediated chemotherapy resistance [103]. Common onco-miRs such as miR-21, miR-155, miR-372, miR-373, and the miR-17–92 cluster promote proliferation, invasion, and chemoresistance [19, 101], whereas tumor-suppressive miRNAs including let-7, miR-34, miR-15a, and miR-16–1 inhibit oncogenic pathways but are often downregulated in cancer [19, 101]. In CLL, deletion or reduced expression of miR-15a/16–1 leads to BCL2 overexpression and leukemogenesis [101]. In CML, miRNA signature panels – including miR-145, miR-151a, miR-185, and miR-628 – correlate with tyrosine kinase inhibitor response and disease stage [104].

Therapeutically, miRNA mimics and inhibitors are under investigation [16] (Table 1), though translation remains challenging. Clinical testing of the miR-34a mimic MRX34 demonstrated tumor localization, off-target distribution and immune-related adverse events (irAEs), resulting in limited responses [105]. These challenges likely contribute to the small number of ongoing interventional trials [60, 69, 106–109].

Circulating miRNAs offer promise as minimally invasive biomarkers due to their stability in blood and resistance to RNase degradation. However, their clinical utility is limited by technical variability in RNA extraction, low abundance, and the need for highly sensitive, standardized profiling methods. Although numerous candidate circulating miRNAs have been identified across tumor types, large-scale validation is still lacking [19, 101].

### CircRNAs

Circular RNAs (circRNAs) are a type of non-coding RNA that has a covalently closed, continuous loop without 5′ caps or 3′ poly(A) tails. This is formed during a process called back-splicing in which upstream splice acceptors join downstream splice donors. The circular structure confers remarkable stability and resistance to exonuclease degradation compared with linear RNAs. CircRNAs are widely expressed in eukaryotic cells and can originate from exonic, intronic or intergenic regions of the genome [110]. In addition to functioning as microRNA sponges and RNA-binding protein (RBP) interactors, they can act as transcriptional regulators, form scaffolds for protein complexes, and in some cases, be translated into functional peptides in a cap-independent manner. They contribute to diverse cellular processes and are increasingly implicated in tumor biology, including the regulation of stemness (e.g., circEcadherin, circPTN), angiogenesis (e.g., circSMARCA5, circSHKBP1) and epithelial-to-mesenchymal transition (EMT) and metastasis (e.g., circSKA3, circTGB6). Moreover, circRNAs can modulate immune responses and therapy resistance mechanisms in cancer (e.g., circFGFR1, circBIRC6) [58, 110]. CircRNAs show high specificity and sensitivity in clinical samples due to their exceptional stability, abundance, and often tissue- and disease-specific expression patterns, highlighting their strong potential as diagnostic and prognostic biomarkers [58] (Table 1).

In summary, RNA-based therapeutic strategies that leverage RNA interference (RNAi) and messenger RNA (mRNA) to target genes involved in tumorigenesis are increasingly recognized as promising treatment approaches. Small interfering RNAs (siRNAs) have been designed to selectively downregulate oncogenes and thus inhibit the expression of cancer-causing proteins. Simultaneously, mRNA vaccines are being investigated for their potential to trigger robust anti-tumor immune responses, offering a novel approach to cancer immunotherapy. The field is rapidly evolving, with new ncRNA biomarkers and therapeutic candidates being identified through high-throughput sequencing and bioinformatics approaches [111–114]. Therefore, RNA-based interventions have the potential to support personalized, mechanism-driven treatments that can specifically modulate gene expression. This provides a versatile and targeted therapeutic avenue for cancer management [115, 116].

### Transcription factor biomarkers of EMT, stemness and metastasis

Transcription factors (TFs) are central gene expression regulators that act through sequence-specific binding to promoters and enhancers. In healthy tissues, they ensure that cells grow, communicate, and respond appropriately to their environment. In cancer, however, this fine-tuned system can be hijacked: TFs become rewired through genetic or epigenetic alterations, fueling oncogenic programs that drive tumor growth, reshape the microenvironment, and promote therapeutic resistance. Abnormal TF activities can further induce EMT and stemness, enabling cancer cells to invade, metastasize, and escape treatment, positioning TFs as compelling biomarkers and therapeutic targets in precision oncology.

Several TF families have emerged as key players in cancer biology and biomarker development (Fig. 1), reflecting their diverse roles across tumor types. The RUNX family is an example of dual functionality. It is involved in fundamental biological development processes and contributes to tumor initiation, progression, and therapy resistance in both hematologic and solid malignancies [117, 118]. Members of the SOX family, including SOX2, SOX4, SOX9, and SOX18, are frequently overexpressed in cancer, driving metastatic traits and often correlated with poor prognosis [119, 120]. Other notable families include the forkhead box (FOX) and STAT proteins, which exhibit context-dependent roles and function as either tumor suppressors or metastasis promoters, depending on the cellular and molecular context [121, 122]. STATs play a pivotal role at the interface of cancer and immunity [122, 123]. Their involvement in immune signaling pathways, together with frequent hyperactivation across diverse cancer types, contribute to malignant phenotypes and influence immunotherapy responses. Consequently, STATs have been proposed as valuable predictive markers of therapeutic outcomes [124–126]. Similarly, members of the HOX and HOXD clusters have been linked to tumor progression and therapy resistance [127], while KLF and ZNF transcription factors are emerging as promising biomarkers in lung and colorectal cancers [128, 129].

Among the transcriptional programs involved in tumor progression, EMT is of particular interest because it confers mesenchymal traits to epithelial cells that enhance motility, invasiveness, and drug resistance. A core group of EMT-TFs comprise Snail (SNAI1/2), Twist (TWIST1/2), and ZEB (ZEB1/2), which repress epithelial markers such as E-cadherin (CDH1) and activate mesenchymal genes like N-cadherin and vimentin [130–135]. These EMT-TFs function within interconnected regulatory networks, interacting with cofactors such as FOXC2, SOX4, SOX9, and PRRX1 to fine-tune EMT states and cellular plasticity [133, 135–137]. Their activation is induced by cancer-cell-intrinsic mechanisms and TME signaling, like transforming growth factor-β (TGF-β), Wnt/β-catenin, or Notch, which promote transcriptional reprogramming and metastatic potential [130, 136, 138, 139]. Importantly, EMT is increasingly recognized as a dynamic spectrum rather than a binary switch: tumor cells can adopt partial or intermediate states that combine epithelial and mesenchymal traits (epithelial-mesenchymal plasticity, EMP). These hybrid phenotypes enhance tumor heterogeneity and adaptability, contributing to resistance and metastatic dissemination [133, 137, 140, 141]. Although these EMT-TFs are established research biomarkers and are occasionally used in clinical pathology for risk assessment, they are not yet part of routine diagnostic workflows. Their prognostic value has been reported across multiple cancer types but remains context-dependent, often requiring integration into multi-marker panels to achieve clinical relevance [142–147]. These challenges are not unique to EMT regulators but reflect broader obstacles in translating TF biology into clinical applications. The complexity of TF networks complicates their use as standalone biomarkers. Tumor heterogeneity and dynamic transcriptional reprogramming can obscure the predictive power of individual TFs across patients and tumor subtypes. To overcome these limitations, integrative multi-omics and transcriptomic approaches are increasingly being applied to unravel TF-centered regulatory networks that correlate with survival, therapeutic response, and disease progression [148–152]. Members of the E2F family have gained particular attention within this system-level framework. Traditionally recognized as master regulators of cell-cycle progression, E2Fs are now acknowledged for their broader impact on tumor biology and prognosis across several malignancies, including breast, prostate, and advanced melanoma [13, 153, 154]. Among them, E2F1 represents a particularly well-characterized example of how a single transcription factor can integrate proliferative, stemness-associated, and microenvironmental programs. In the following section, E2F1 is therefore discussed as a representative case study illustrating how tumor-intrinsic transcriptional regulators can function as systems-level biomarkers. Consistent with this concept, E2F1 emerges as a pivotal transcriptional hub that connects proliferative, invasive, and immune-modulatory programs [155, 156]. Beyond its canonical role in cell-cycle control, E2F1 regulates key EMT drivers, such as ZEB1 and SNAIL [155, 157], integrating proliferative and plasticity-related signaling to promote tumor adaptability, stemness, metastasis and therapy resistance.

### Cancer stem cell states as tumor-intrinsic biomarkers of plasticity, resistance, and metastasis

CSCs represent a heterogeneous and plastic tumor cell state characterized by self-renewal capacity, multilineage differentiation potential, and enhanced tumor-initiating ability. Instead of constituting a fixed subpopulation, CSCs arise through transcriptional and epigenetic reprogramming driven by oncogenic and stemness-specific TFs such as MYC, OCT4, SOX2, and NANOG (Yamanaka factors), as well as signaling pathways including Wnt/β-catenin, PI3K/AKT, and JAK–STAT [158–160]. This plasticity enables non-stem tumor cells to acquire stem-like properties in response to therapy or microenvironmental stress, positioning CSCs as functional readouts of tumor-intrinsic regulatory programs.

CSCs are typically maintained within specialized stem cell niches composed of stromal cells, extracellular matrix components (ECM), hypoxic gradients, and soluble factors that reinforce stemness and protect against immune and therapeutic pressures [158]. Hypoxia-inducible transcriptional programs stabilize CSC phenotypes by coordinating metabolic adaptation, epithelial–mesenchymal plasticity, and sustained pluripotency factor expression. At the epigenetic level, aberrant DNA methylation and imbalanced histone modifications further stabilize CSCs in permissive chromatin states that support lineage plasticity and therapy resistance [16, 160].

CSC phenotypes are reflected by the expression of stemness-associated proteins and surface markers, including CD44, CD133, and ALDH1, which correlate with metastatic competence, drug resistance, and disease relapse across multiple cancer types [161]. Notably, E2F1 directly activates NANOG transcription through promoter binding, linking cell-cycle-associated transcriptional networks to CSC maintenance and providing a mechanistic bridge between proliferative and stem-like tumor states [6, 162]. This connection positions CSC biology as a natural conceptual transition toward E2F-centered transcriptional programs.

From a biomarker perspective, CSC-associated transcriptional and epigenetic signatures have been detected both within tumor tissues and in circulating tumor-derived compartments. A subset of CTCs exhibits stem-like features and contributes disproportionately to metastatic seeding and therapy resistance [163]. While not all CTCs derive from CSCs, the presence of stem-like CTCs has been associated with poor prognosis and early metastatic dissemination [164, 165]. However, clinical use remains challenging due to phenotypic heterogeneity, EMP, and technical limitations in detection [166].

Collectively, CSCs function as integrative tumor-intrinsic biomarkers that capture transcriptional, epigenetic, and phenotypic plasticity underlying metastasis, drug resistance, and relapse. Their regulation by core oncogenic TF networks places CSC-associated programs at the intersection of stemness, EMT, and cell-cycle control, providing a mechanistic and conceptual link to downstream transcriptional regulators such as E2F family members.

### E2F transcription factors as cancer biomarkers and emerging therapeutic targets

Activation of E2F family members is essential for cell-cycle control, differentiation, proliferation and the DNA-damage response [6, 167]. E2F1–3a generally act as transcriptional activators, whereas E2F3b/4–8 function mainly as repressors [13, 168–170]. Several E2F members are dysregulated or mutated in cancer, and high expression of E2F1/2/4/5/6 has been reported across multiple tumor types (melanoma, breast, stomach adenocarcinoma, nasopharyngeal and gastric cancers) compared with normal tissue [13, 169–171]. E2F levels are frequently higher in metastatic versus primary lesions and associate with worse outcomes; they also correlate with neoantigen burden from SNVs, CNAs and indels [13, 172]. In ER-positive/HER2-negative breast cancer, a gene expression-based E2F pathway score that was derived from the hallmark E2F targets gene set, was associated with a higher pathological complete response (pCR) rate following neoadjuvant chemotherapy. A high E2F pathway score was also strongly correlated with sensitivity to cyclin-dependent kinase (CDK) inhibitors in cell lines, further supporting its potential as a predictive biomarker [13]. Accordingly, CDK4/6 inhibitors such as palbociclib have improved patient outcomes [173]. In addition, CDK2 inhibitors such as PF-07104091, which is currently in clinical trials (NCT05262400, NCT04553133), can delay E2F activation, reflecting the cooperative regulation of E2F by CDK2 and CDK4/6 [174].

### E2F1 is a driver of metastasis and tumor microenvironmental reprogramming

E2F1 is highly expressed across many solid tumors (skin, breast, liver, lung, kidney, ovarian, cervical, uterine, colorectal, melanoma, bladder, gastric, pancreatic, prostate, and head-and-neck cancers) and is a central regulator of invasion and the metastatic cascade as well as reprogramming of the tumor immune microenvironment [6, 155, 156, 168–170, 172, 175–177]. Although E2F1 initially functions in tumor surveillance by promoting apoptosis after DNA damage, it can switch during progression to drive chemoresistance, angiogenesis, extravasation, and EMT, thereby promoting metastasis in preclinical models [97, 153, 155, 178, 179]. Elevated E2F1 expression is associated with poorer outcomes in multiple cancer types [6, 168–170, 172, 177, 180–186]. In melanoma, E2F1 promoter hypomethylation correlates with increased expression, implicating epigenetic derepression in melanomagenesis [170]. In contrast, hypermethylation of specific E2F1-associated CpG sites has been linked to poorer survival without a consistent association with total E2F1 expression, indicating a potential prognostic rather than transcriptional role [177]. Genetic alterations in *E2F1* occur in approximately 10% of melanoma cases [170], and E2F1 expression is higher in metastatic than in primary tumors [172]. Increased E2F1 activity has been linked to melanoma invasion and metastatic progression, in part through its role in maintaining cancer cell stemness [177, 187]. Consistently, E2F1 target genes are abundantly expressed in metastatic tissues and promote EMT, migration, and invasion [170]. Functionally, E2F1 has also been correlated with multidrug resistance (MDR) and is predictive of chemoresistance and lymphogenic metastasis in penile cancer [188, 189].

E2F1 overexpression in cancer cells also alters their interaction with the TME by promoting a pro-inflammatory milieu [156, 177]. We previously showed that the E2F1-driven melanoma secretome activates an IL-6-based gene regulatory network in T cells, reshaping the transcriptional landscape and cytokine profile of CD4^+^ T cells toward a Th2-polarized state [156], consistent with observations in melanoma patient samples [177]. Elevated E2F1, STAT3, and IL-6, factors linked to stemness and tumor progression, correlate with increased Th2 infiltration and suppression of Th1 responses in both primary and metastatic melanoma. Conversely, depletion of these mediators restores Th1/Th2 and promotes a Th1-dominant antitumor immunity. Tumor-intrinsic E2F1 also alters CD8^+^ T cell transcriptional and cytokine profiles, including enhanced IL-6 secretion. Because IL-6-STAT3 signaling inhibits differentiation of cytotoxic CD8^+^ lymphocytes and impairs PD-L1 blockade during immune checkpoint therapy, these findings support a central role for E2F1 in attenuating T cell function and fostering immunotherapy resistance [156].

### Transcriptional coregulators of E2F1

Overexpression of E2F1 drives cancer progression and immune modulation through its transcriptional coregulators (coTFs), many of which are themselves E2F1 target genes and form protein complexes that enhance metastatic programs such as angiogenesis, extravasation, and invasion [190–192]. For example, the E2F1–MTA1 complex promotes lung metastasis by upregulating HAS2 and hyaluronic acid (HA), which recruit immunosuppressive M2-type TAMs. Disrupting this interaction with argatroban in melanoma and pancreatic cancer models reduces HAS2/HA production, prevents formation of a pro-metastatic TME and avoids cancer relapse [193]. Moreover, E2F1 also directly interacts with IL-6-activated STAT3 to drive IL-6 transcription via an E2F1/STAT3-responsive promoter element [156].

Retinoblastoma protein (Rb) is another classical E2F1 regulator. Hypophosphorylated Rb binds and suppresses E2F1, whereas CDK-mediated phosphorylation disrupts this complex and activates E2F1. Preventing Rb–E2F1 dissociation with arsenic trioxide decreases E2F1 activity [194, 195]. Clinically, arsenic trioxide, which is used as FDA-approved therapy for acute promyelocytic leukemia (APL), induces complete remission when combined with all-trans retinoic acid (ATRA) and results in lower hematologic toxicity than standard ATRA plus chemotherapy [196]. However, dosage and treatment duration must be carefully managed due to arsenic-associated toxicities [197, 198]. Mechanistically, arsenic trioxide increases Rb expression and alters its phosphorylation at T373 and S608, thereby reducing E2F1 and cyclin E levels [194, 195]. Consistent with this, therapeutic strategies aimed at maintaining Rb in a hypophosphorylated, E2F1-repressive state are being explored [174].

### E2F1 as a prognostic biomarker and therapeutic target

Therefore, E2F1 has gained substantial interest as a predictive [189], prognostic [6, 168–170, 177, 185, 186], and therapeutic biomarker [168, 171, 172, 184, 199]. Silencing E2F1 with siRNA suppresses tumor growth in multiple cancer models [171, 172, 177, 186, 200] and reduces macrophage and dendritic-cell infiltration in the TME in vivo [171, 193]. Despite the therapeutic promise, achieving cancer-specific on-target inhibition remains a major challenge.

The E2F1/4 inhibitor HLM006474—well tolerated by normal melanocytes and keratinocytes—has shown encouraging effects in mouse models [172, 177]. Although E2F1 and E2F4 overexpression is correlated with poor overall survival in gastric cancer [168], only E2F1 predicts adverse outcomes in metastatic melanoma [172]. Mechanistically, HLM006474 suppresses melanoma proliferation by inhibiting E2F1 target genes such as *BRCA1*, *BIRC5*, and *CDC6*, thereby inducing G2/M arrest and activating apoptosis and senescence pathways via caspase-3 and p53 [172]. To date, E2F1-targeted approaches have been primarily tested in p53-wild-type models, and all therapeutic investigations remain preclinical.

The main obstacles to clinical translation are specificity and avoidance of off-target toxicity, given E2F1’s physiological roles in normal cells. Potential strategies include delivering E2F1 inhibitors in vesicles targeting cancer-specific surface markers, or restricting inhibitor activation to oncogenic contexts, such as hTERT-, Twist1-, or survivin-expressing cells that are inactive in healthy adult tissues. Such approaches may enable the selective targeting of E2F1-driven tumors while minimizing the harm to normal tissues.

## Immune-related molecular biomarkers in cancer

The progression of cancer and the response to therapy are significantly influenced by tumor-driven reprogramming of the immune landscape via transcriptional and epigenetic mechanisms. The expression of cytokines, chemokines, immune checkpoint molecules, and antigen-presentation machinery is determined by oncogenic signaling pathways, lineage-defining transcription factors, and chromatin-based regulatory processes that primarily operate within cancer cells and their associated stromal compartments. These regulatory programs shape immune signaling, inflammatory tone, and checkpoint activation within the tumor milieu. In this context, immune-related biomarkers should be regarded as downstream molecular readouts of integrated gene regulatory networks rather than as isolated immune parameters. Cytokines occupy a central position within these networks, acting as functional mediators of tumor-immune crosstalk and system-level indicators of underlying transcriptional states. Consequently, clinically applied immune biomarkers, including immune checkpoint molecules, mutational burden-associated metrics, and antigen-based metrics, reflect the transcriptional and epigenetic programs that govern immune evasion, tumor plasticity, and therapeutic vulnerability to varying extents.

### Cytokines in cancer: biological roles and biomarker potential

Cytokines are small, secreted proteins that mediate communication between cancer cells, stromal components, and immune cells. They orchestrate inflammation, immune surveillance, angiogenesis, and tissue remodeling, and are central to how tumors reshape their microenvironment. Cytokine networks are frequently rewired in cancer, leading to chronic inflammatory signaling, immune suppression, and support of tumor growth and metastasis. Because many cytokines are released into blood or other body fluids, they are attractive candidates for minimally invasive biomarkers reflecting tumor burden, aggressiveness, and immune landscape [201–203].

Among the most consistently reported cytokine biomarkers, interleukin-6 (IL-6) has emerged as a central node. Elevated IL-6 is observed across multiple solid tumors, including breast, lung, colorectal, gastric, pancreatic, and hepatocellular carcinoma, and correlates with advanced stage, cachexia, therapy resistance, and poor survival [203–206]. Importantly, high baseline IL-6 level predict inferior outcomes in patients receiving immune checkpoint inhibitors (ICIs), supporting its use as a negative predictive biomarker in immunotherapy [207–209].

IL-8 (CXCL8) is another robust marker associated with tumor progression, angiogenesis, and metastasis. Elevated circulating IL-8 predicts poor prognosis and resistance to targeted therapies and ICIs, particularly in melanoma, lung, and gastric cancers [205, 207, 209, 210]. Tumor necrosis factor-α (TNF-α), which was originally defined as an anti-tumor cytokine, is chronically elevated in many cancers and supports tumor-promoting inflammation, EMT, and immune dysfunction; increased TNF-α levels correlate with poor outcomes in breast, oral, and gastrointestinal cancers [203, 211, 212].

Immunosuppressive cytokines, such as IL-10 and TGF-β are frequently increased in advanced disease and are associated with immune evasion and worse prognosis [201, 203, 207]. Conversely, higher levels of IL-2 and interferon-γ (IFN-γ) sometimes correlate with improved responses to ICIs, reflecting a more active anti-tumor immune milieu [207, 213, 214].

Disease-specific applications highlight both the promise and limitations. In oral squamous cell carcinoma, salivary IL-6, IL-8, TNF-α, and IL-1β repeatedly show high diagnostic accuracy, supporting cytokines as non-invasive early detection tools [211, 215–217]. In colorectal and gastric cancer, panels including IL-6, IL-8, IL-17A, and IL-33 correlate with tumor stage and prognosis [203, 218, 219]. In lung cancer, multiplex serum cytokine signatures outperform single analytes for predicting survival and immunotherapy benefit [205, 207, 208].

Multiplex “cytokinome” profiling is increasingly emerging as a more informative strategy than single-cytokine measurements. Panels better capture the complexity of tumor–immune interactions and improve diagnostic and predictive performance, particularly in the context of immunotherapy [202, 207, 220]. However, pleiotropy, overlapping with non-cancer inflammation, and lack of standardized assays continue to limit routine clinical implementation [201–203, 206, 221].

### Transcriptional and epigenetic control of cytokine networks in cancer

Cytokine dysregulation in cancer is not random but arises from coordinated transcriptional and epigenetic reprogramming driven by oncogenic signaling and microenvironmental cues. Central inflammatory TFs integrate these signals and establish cytokine expression programs (Table 2) that shape tumor progression and immune landscapes.

**Table 2:** Overview of important transcription factor families controlling cytokine programs in cancer

TF/TF family	Role in cytokine control	Ref.
NFκB	Drives TNF, IL1, IL6, chemokines; central inflammatory hub in TME	[222–224]
STAT3/STAT1/STAT5	Mediators of IL6family, IFNs, other cytokines; promote inflammatory and survival programs	[225, 226]
AP1 (FOS/JUN)	Cytokineresponsive enhancer TF, coregulates inflammatory genes	[223, 227]
Smads (TGFβ)	Transduce TGFβ; cooperate with NFκB/STAT3 and EMTTFs in cytokinedriven EMT	[228]
Snail, Twist, Zeb	EMTTFs induced by inflammatory cytokines, help maintain inflammatory/CSC networks	[228]
GLI2	Required for CCL5induced IL6 expression in stromal cells	[229]
E2F1 + STAT3	Autocrine IL6 loop, reshapes Th1/Th2 cytokine milieu in melanoma	[156]
NFAT/TOX/NR4A	Exhaustion program, suppresses effector cytokines in tumorinfiltrating T cells	[230]

NF-κB is a master regulator of cancer-associated inflammation and directly controls transcription of IL-1β, IL-6, TNF-α, and multiple chemokines. Constitutive NF-κB activation in tumor and stromal cells links chronic inflammation with carcinogenesis, angiogenesis, and immune suppression [222, 228]. STAT family members, particularly STAT3, are activated downstream of IL-6 and IL-10 and reinforce cytokine loops by inducing further IL-6 expression, cancer stemness, and immune-evasive programs [228, 231]. These NF-κB–STAT3 feed-forward circuits exemplify how tumors stabilize the inflammatory cytokine milieus.

Additional TFs such as AP-1, C/EBPβ, CREB, and interferon regulatory factors (IRFs) cooperate at cytokine promoters and enhancers, often under epigenetic control, to fine-tune inflammatory outputs [228, 232, 233]. In TAMs, C/EBPβ and microRNA-155 regulate high-level cytokine production, illustrating how epigenetic and post-transcriptional mechanisms sculpt cytokine secretion [232]. In T cells, exhaustion-associated TFs such as TOX, NR4A, and NFAT5 suppress IL-2, IFN-γ, and TNF expression, imprinting dysfunctional immune states that limit anti-tumor immunity [230, 234].

Oncogenic cofactors further connect transcriptional control with therapeutic vulnerability. The cytoplasmic tail of MUC1 forms complexes with NF-κB on cytokine promoters to enhance IL-6 and TNF-α transcription in breast cancer [235]. Nuclear focal adhesion kinase (FAK) regulates IL-33 and CCL5 transcription through chromatin-associated networks, promoting immune evasion in squamous carcinomas [236]. These examples underscore how cancer-specific transcriptional assemblies drive distinct cytokine programs.

Clinically, these regulatory axes are already being targeted. JAK/STAT inhibitors, NF-κB-modulating agents, and epigenetic drugs can indirectly normalize cytokine landscapes, whereas direct cytokine-centered strategies (e.g., IL-6, TGF-β blockade) are under active clinical investigation, often in combination with ICIs [207, 237]. Integrating cytokine biomarkers with transcriptional and epigenomic signatures may therefore enable not only improved patient stratification but also rational selection of cytokine-modulating therapies.

### TGF-β signaling in cancer progression and the tumor microenvironment

TGF-β is a multifaceted cytokine that plays a critical role in regulating diverse processes, such as cell proliferation, differentiation, apoptosis, and immune homeostasis. Canonical TGF-β signaling is initiated by ligand binding to type I and type II TGF-β receptors, leading to activation of SMAD2/3, complex formation with SMAD4, and transcriptional regulation of target genes. In normal epithelia and early tumorigenesis, this pathway enforces cytostatic and tumor-suppressive programs. During cancer progression, however, genetic and epigenetic alterations enable tumor cells and stromal compartments to exploit TGF-β signaling to promote invasion, metastasis, and immune evasion [238, 239].

**Fig. 2:**
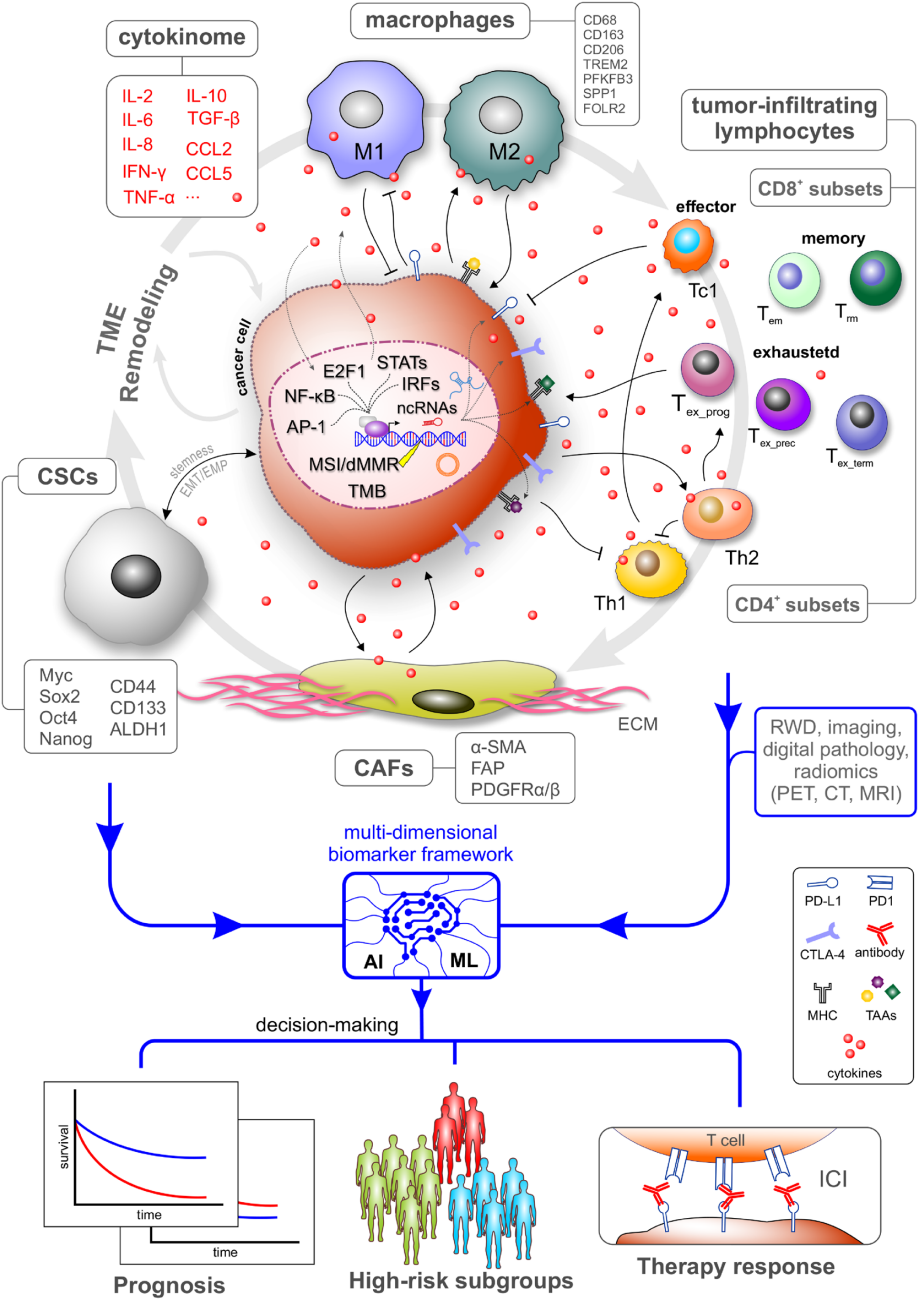
AI-based multi-dimensional biomarker framework. The integration of tumor-intrinsic regulators with the cellular and spatial characteristics of the TME enhances the explanatory power of biomarkers, improves their contextual robustness, and augments their predictive value. This perspective facilitates the reconciliation of inconsistencies observed across single-marker studies and elucidates the rationale behind the cancer-type – and context-dependent performance of biomarkers. Recent technological advancements in multi-omics profiling, spatial technologies, and AI/ML-driven integration have rendered the realization of this regulatory framework feasible. These advancements enable the development of composite biomarkers that more effectively capture tumor plasticity and immune dynamics

In advanced cancers, TGF-β drives EMT through cooperation between SMAD complexes and other transcription factors, including ZEB, SNAIL, and TWIST family members, thereby enhancing motility, invasiveness, and stem-like traits. TGF-β also profoundly remodels the TME, activating fibroblasts into cancer-associated fibroblasts, stimulating ECM deposition, promoting angiogenesis, and skewing immune infiltrates toward immunosuppressive phenotypes such as regulatory T cells and M2-like macrophages (Fig. 2). These transcriptionally controlled programs establish a pro-tumorigenic niche that supports metastatic dissemination and therapeutic resistance [130, 239, 240].

### TGF-β–E2F1 crosstalk: context-dependent reprogramming of transcriptional networks

TGF-β provides a representative example of a cytokine-driven regulatory network that is extensively repurposed during cancer progression to promote cellular plasticity, immune evasion, and stromal remodeling. In addition to its canonical SMAD-dependent transcriptional activity, TGF-β reshapes broader gene regulatory programs through interactions with other transcription factors such as E2F1 [155].

In normal epithelial cells and early tumor stages, TGF-β enforces cytostasis in part by suppressing canonical E2F1 activity. TGF-β signaling reduces G1 cyclin-dependent kinase activity, maintains RB in a hypophosphorylated state, and sequesters E2F factors away from S-phase promoters, resulting in repression of DNA replication and cell-cycle genes [241, 242]. TGF-β further decreases E2F1 mRNA and protein levels, and forced expression of E2F1 can override TGF-β-induced growth arrest, underscoring E2F1 as a critical downstream effector of cytostatic signaling [241]. Importantly, TGF-β can qualitatively alter E2F1 function at selected promoters: under growth-inhibitory conditions, E2F1 cooperates with RB to repress targets such as EBP1, illustrating that TGF-β can switch E2F1 from an activator to a repressor in a promoter- and context-dependent manner [243].

During tumor progression, this regulatory axis is reprogrammed. Rather than restraining proliferation, TGF-β redirects E2F1-dependent transcription toward EMT- and invasion-associated outputs. In prostate cancer, E2F1 drives expression of the miR-20b-5p precursor, which targets TGFBR2. TGF-β signaling suppresses both E2F1 and miR-20b-5p, relieving repression of TGFBR2 and establishing a feed-forward loop that sustains EMT signaling. Perturbation of this circuit alters cadherin switching, vimentin expression, and invasive capacity, demonstrating that TGF-β repurposes E2F1 activity from cell-cycle regulation toward modulation of EMT and metastatic traits [244]. Collectively, these findings position E2F1 as a transcriptional rheostat through which TGF-β signaling suppresses classical proliferative programs in early stages but fine-tunes EMT and invasion programs in advanced disease [130, 238, 240, 245].

### TGF-β as a biomarker and therapeutic target in cancer

TGF-β, particularly the TGF-β1 isoform, is widely investigated as a diagnostic and prognostic biomarker for multiple tumor types, including hepatocellular carcinoma and metastatic colorectal cancer. Elevated TGF-β levels in tumor tissue or circulation are frequently correlated with poor prognosis, increased tumor aggressiveness, metastatic burden, and resistance to chemotherapy and immunotherapy [231, 246–249]. TGF-β pathway signatures also associate with immune-excluded or immunosuppressed TMEs, supporting their potential utility in predicting immune checkpoint blockade responses and guiding patient selection for TGF-β-targeted interventions [247, 250]. However, the dual role of TGF-β as a tumor suppressor in early lesions and a tumor promoter in advanced disease complicates its use as a universal biomarker. Its clinical utility will likely depend on context-aware biomarker strategies integrating TGF-β levels with transcriptional, stromal, and immune signatures that reflect pathway output rather than ligand abundance alone [246, 251].

Therapeutically, the TGF-β pathway is a major focus of drug development. Multiple approaches are under clinical investigation, including small-molecule inhibitors of TGF-β receptor kinases (e.g., galunisertib), monoclonal antibodies and ligand traps that neutralize TGF-β, and antisense oligonucleotides or vaccine-based strategies that modulate pathway activity [248, 250, 252, 253]. These agents are being tested across hepatocellular, lung, breast, prostate, colorectal, and brain cancers. While preclinical studies have demonstrated robust antitumor and immunomodulatory effects, clinical translation has been challenging due to context-dependent biology and treatment-related toxicities, including immunosuppression and cardiovascular adverse events [250, 251, 254, 255].

Current strategies increasingly emphasize rational combinations, particularly with ICIs, and biomarker-guided patient selection to maximize therapeutic benefit while minimizing risk. In this setting, TGF-β functions not only as a therapeutic target but also as a dynamic biomarker reflecting transcriptional programs that govern tumor plasticity, immune escape, and metastatic competence.

While cytokines and cytokine-driven transcriptional networks provide mechanistic insight into how tumor–immune interactions are established and maintained, most clinically approved immune biomarkers capture only selected downstream manifestations of these regulatory programs. Immune checkpoint expression, mutational burden-related metrics, and antigen-based markers therefore represent partial and context-dependent readouts of tumor-intrinsic signaling, TF activity, and epigenetic regulation. As a result, their predictive performance varies across tumor types and disease stages, reflecting differences in transcriptional state, immune cell composition, and spatial organization within the tumor milieu. Thus, interpreting these biomarkers in the context of upstream regulatory networks is essential for understanding their limitations and advancing biomarker-guided immunotherapy.

### PD-L1

Immune evasion represents a central consequence of tumor-driven transcriptional and epigenetic reprogramming, enabling cancer cells to suppress antitumor immune responses through coordinated modulation of immune checkpoint pathways. Among these, programmed death-ligand 1 (PD-L1) is a key inhibitory molecule whose expression on tumor and stromal cells attenuates T cell and natural killer cell function through engagement of the PD-1 receptor [14, 256]. Therapeutic blockade of the PD-1/PD-L1 axis has produced durable clinical benefit in subsets of patients with highly immunogenic malignancies like melanoma, renal cell carcinoma, and non-small cell lung cancer (NSCLC), leading to its widespread clinical implementation.

However, PD-L1 expression represents a dynamic and context-dependent output of tumor-intrinsic signaling, cytokine exposure, transcription factor activity, and epigenetic regulation, rather than a stable determinant of immune responsiveness. Consequently, PD-L1 immunohistochemistry has shown limited and variable predictive performance across tumor types and disease stages, despite its use for patient stratification in ICI regimens [257, 258].

At the molecular level, PD-L1 expression is governed by the integration of inflammatory and oncogenic transcriptional programs layered onto the epigenetic state of the *CD274* locus. Cytokine-driven induction is primarily mediated through the IFNγ–JAK–STAT1–IRF1 axis, with IRF1 binding defined interferon-responsive elements in the PD-L1 promoter, while STAT3 contributes to PD-L1 upregulation downstream of IL-6 and oncogenic JAK/STAT signaling [259–261]. In parallel, constitutive or stress-induced PD-L1 expression is promoted by NF-κB, AP-1, MYC, hypoxia-inducible factors, and EMT-associated TFs such as ZEB1, linking inflammatory cues, hypoxia, oncogenic signaling, and EMP to immune evasion [262–265].

Crucially, TF access and activity at the PD-L1 promoter are constrained by the chromatin context. DNA hypomethylation and loss of repressive histone marks (H3K9me3/H3K27me3), together with enrichment of activating H3K4me3, create a permissive chromatin environment that enables inducible and oncogenic TF programs to drive PD-L1 expression, whereas repressive chromatin states restrict PD-L1 responsiveness despite inflammatory signaling [266–269]. Epigenetic regulators such as EZH2, DNMTs, and BRD4 further modulate PD-L1 output by controlling both *CD274* accessibility and the expression of key transcriptional activators, including IRF1 and NF-κB, thereby contributing to inter- and intratumoral heterogeneity of PD-L1 expression [261, 270, 271].

Consistent with its context-dependent regulation, PD-L1 expression demonstrates predictive value in only a minority of ICI approvals, with PD-L1 status being predictive in approximately 30% of FDA approvals across tumor types [272]. In some settings, combinatorial approaches modestly improve predictive performance; for example, concurrent high PD-L1 expression and elevated tumor mutational burden (TMB) correlate with improved outcomes in subsets NSCLC patients treated with PD-1/PD-L1 inhibitors [273]. However, this association is not generalizable across malignancies, as similar biomarker combinations fail to predict response in gastric and esophageal adenocarcinomas [274]. These discrepancies reflect the dynamic and multilayered regulation of PD-L1, influenced by tumor stage, anatomical context, treatment history, and environmental factors such as smoking [275, 276]. In addition, post-translational modification of PD-L1, particularly N-linked glycosylation, can mask epitopes and lead to false-negative IHC results. Accordingly, analysis of de-glycosylated PD-L1 significantly improves predictive accuracy in triple-negative breast cancer and other settings [261, 277, 278].

Importantly, disruption of epigenetically maintained immune-evasion programs using DNA methyltransferase inhibitors can enhance responsiveness to PD-1/PD-L1 blockade, in part by restoring antigen presentation and cytotoxic T cell activity [279]. Together, these observations underscore that PD-L1 expression reflects only one dimension of a broader regulatory landscape and should be interpreted in conjunction with genomic, epigenetic, immune, and clinical parameters for effective patient stratification.

### Tumor mutational burden

TMB is defined as the total number of somatic mutations, including insertions, deletions, and substitutions, per megabase of tumor DNA [280, 281]. TMB has been proposed as a surrogate measure of neoantigen load, based on the premise that a higher number of somatic mutations increases the probability of generating immunogenic TSAs [280]. However, accumulating evidence indicates that less than 5% of somatic mutations ultimately give rise to immunogenic neoantigens, underscoring that mutational burden alone does not equate to functional tumor immunogenicity [5].

This limited translation reflects the requirement for somatic mutations to be transcriptionally expressed and for their encoded peptides to be efficiently processed and presented via major histocompatibility complex (MHC) molecules, processes that are tightly regulated at both the transcriptional and epigenetic level and depend on intact antigen-processing machinery. Tumors frequently suppress the expression of MHC genes and associated antigen-processing components through transcriptional silencing, epigenetic modifications, or genetic alterations, thereby uncoupling mutational burden from effective immune recognition [282–286]. Key regulators of antigen presentation, including NLRC5 for MHC class I and CIITA for MHC class II, as well as components such as TAP1/2, β2-microglobulin, and immunoproteasome subunits, are themselves subject to transcriptional and epigenetic control and are frequently downregulated in cancer, further limiting neoantigen display despite high mutational load [284, 286–289].

Consistent with these constraints, the predictive value of TMB varies markedly across cancer types and clinical contexts. High TMB (TMB-H), commonly defined as ≥ 10 mutations per megabase, is more strongly associated with benefit from ICI in malignancies such as NSCLC and melanoma, whereas its predictive performance is substantially lower or inconsistent in other tumor types, including cervical, breast, and prostate cancers [280, 281]. In a large pan-cancer analysis of patients treated with anti-PD-1 therapy, only 29% of TMB-H tumors exhibited an objective response, highlighting the limited utility of TMB as a standalone biomarker [280].

The predictive capacity of TMB can be modestly improved in selected settings using combinatorial biomarker strategies. For example, concurrent high TMB and elevated PD-L1 expression correlate with improved clinical outcomes in subsets of patients with NSCLC receiving PD-1/PD-L1 blockade [273]. However, this association does not generalize across malignancies, as similar biomarker combinations fail to reliably predict response in gastroesophageal adenocarcinoma and other tumor types [274]. Such discrepancies likely arise from heterogeneity in tumor stage, spatial architecture, clonal composition, immune infiltration, and dynamic changes during disease progression and treatment, as well as from technical variability in TMB assessment methodologies and threshold definitions [274, 276, 281].

Beyond mutational load alone, multiparametric approaches incorporating immune contexture further refine predictive power. Tumors with high TMB, accompanied robust tumor-infiltrating CD8^+^ lymphocytes and an increased neoantigen burden, such as melanoma, lung, and bladder cancers, are more likely to respond to immune checkpoint blockade, whereas tumors lacking this coordinated immune-genomic profile, including glioma, breast, and prostate cancers, typically exhibit limited benefit [5, 14, 290].

### Microsatellite instability (MSI) & DNA mismatch repair deficiency (dMMR)

Microsatellite instability (MSI) reflects hypermutability arising from defects in the DNA mismatch repair (MMR) system, which normally preserves genomic integrity by correcting base–base mismatches, insertion–deletion loops, and misincorporated nucleotides generated during DNA replication and recombination [291]. Deficient mismatch repair (dMMR) can result from germline or somatic mutations in MMR genes or from epigenetic silencing through promoter hypermethylation, leading to functional MMR heterodimer destabilization and loss of nuclear protein expression [291]. Impaired MMR activity promotes the accumulation of replication errors and genomic instability, which frequently manifest as MSI and contribute to increased TMB [292, 293].

High MSI (MSI-H) and dMMR are therefore clinically relevant biomarkers in oncology. They are widely used for patient stratification in immunotherapy, particularly in ICI treatment [5, 294]. However, the prevalence of dMMR/MSI varies markedly across solid tumor types, occurring in approximately 20–40% of endometrial cancers but in only ~4% of other solid malignancies [294]. MSI-H/dMMR status is especially important for identifying subsets of colorectal carcinomas and guiding immunotherapy decisions, although optimal thresholds for immune checkpoint blockade across different cancer entities remain incompletely defined [15, 293].

Despite its clinical utility, biological and technical heterogeneity limit the application of dMMR/MSI as a standardized biomarker. Diagnostic assessment typically relies on IHC, PCR-based assays, or next-generation sequencing targeting the four core MMR proteins MLH1, PMS2, MSH2, and MSH6, yet concordance between methods varies substantially outside colorectal and endometrial cancers [294]. Large in silico analyses encompassing more than 24,000 patients across diverse solid tumors have revealed pronounced heterogeneity in MMR gene alterations, with MSH6 emerging as the most frequently mutated gene but not consistently associated with MSI or dMMR phenotypes [294]. Notably, tumors harboring pathogenic MMR gene mutations exhibit MSI in only approximately half of cases, and microsatellite-stable tumors may still carry MMR-related mutations, indicating that MMR gene alterations and MSI status are related but not synonymous [294].

Beyond genomic instability, the immunogenic consequences of dMMR/MSI are strongly shaped by transcriptional programs and epigenetic states that regulate antigen expression, processing, and immune cell recruitment. Although MSI-H tumors accumulate frameshift mutations capable of generating abundant neoantigenic peptides, effective immune recognition requires transcriptional expression of mutant genes and intact antigen-processing and presentation machinery, which are frequently disrupted in MSI-H cancers [295–297]. Large colorectal cancer cohorts have demonstrated recurrent genetic and functional alterations in antigen-presentation components, including HLA class I molecules, β2-microglobulin, TAP1/2, NLRC5, and RFX5, resulting in reduced MHC-I surface expression and immune escape despite high neoantigen load [296, 298, 299].

In parallel, epigenetic mechanisms further restrict immune visibility in MSI-H tumors. dMMR itself frequently arises through epigenetic silencing of MLH1. Broader epigenetic reprogramming, which encompasses DNA methylation, histone modifications, and miRNA-mediated regulation, can suppress antigen-presentation genes, interferon signaling, and chemokine expression, thereby reshaping the TME toward immune exclusion or dysfunction [300–303]. These transcriptional and epigenetic layers generate pronounced immune heterogeneity among MSI-H tumors, giving rise to “immune-high” and “immune-low” subsets with distinct T cell infiltration, exhaustion states, and responses to immune checkpoint blockade, despite comparable mutational and neoantigen burdens [298, 304–306].

In this context, MSI-H/dMMR represents an established, FDA-approved pan-cancer biomarker for PD-1 blockade and a cornerstone of immunotherapy stratification in colorectal and gastric cancers [307–311]. However, accumulating evidence indicates that therapeutic benefit depends not only on MSI status itself, but on the qualitative immune phenotype of MSI-H tumors, including antigen-presentation competence, immune gene expression programs, and TME composition [307, 312, 313]. Accordingly, transcriptional immune signatures and epigenetic features, such as *MLH1* promoter methylation and chromatin states affecting antigen-presentation and immune-modulatory genes, are emerging as refinement biomarkers to explain non-response and guide patient stratification beyond binary MSI classification [307, 309, 311]. Future biomarker strategies are therefore converging on integrative models that combine MSI with transcriptional, epigenetic, and dynamic immune readouts to more accurately predict immunotherapy response [258, 308, 314, 315].

### Tumor-associated antigens

Tumor-associated antigens (TAAs) and tumor-specific antigens (TSAs) arise from aberrant gene expression programs in cancer cells and are minimally expressed or absent in most healthy adult tissues. Their expression frequently reflects lineage identity, differentiation state, or oncogenic transcriptional and epigenetic deregulation. Accordingly, TAAs have long been explored as biomarkers for cancer detection, disease monitoring, prognosis, and therapeutic stratification, as well as targets for immunotherapeutic intervention.

Clinically, circulating TAAs such as alpha-fetoprotein (AFP), carcinoembryonic antigen (CEA), cancer antigen 12–5 (CA125), CA19-9, and PSA are widely used for diagnosis, treatment monitoring, and recurrence detection based on altered serum levels [316–318]. However, when applied individually, their diagnostic sensitivity and specificity remain limited due to expression in non-malignant tissues and inter- and intratumoral heterogeneity [319, 320]. To improve performance, combinatorial strategies such as multiplex TAA panels and autoantibody signatures have been developed, acting as immunologic reporters of underlying oncogenic pathways and significantly enhancing diagnostic accuracy [316, 318]. For example, combined assessment of CEA, CA125, CA19-9, and gastrin-17 improves diagnostic specificity in gastric cancer compared with single-marker approaches [319].

Beyond soluble biomarkers, cellular TAAs can serve as sensitive indicators of disease burden and therapeutic response. Wilms’ tumor 1 (WT1) exemplifies this dual role, functioning both as a biomarker for minimal residual disease and as an immunotherapeutic target in AML and myelodysplastic syndromes. *WT1* transcript levels correlate with clinical outcomes. Elevated levels predict relapse and subsequent mortality, whereas reduced expression is associated with improved survival. This may reflect enhanced WT1-specific CD8^+^ T cell responses in patients who achieve durable remission [321]. Consequently, WT1-directed strategies, including peptide vaccination and adoptive T cell therapies, are currently under active clinical investigation [321, 322].

At a mechanistic level, TAAs are not a discrete molecular class but represent downstream readouts of oncogenic transcriptional and epigenetic reprogramming. Many clinically relevant TAAs, particularly cancer-testis antigens (CTAs), are normally silenced by DNA methylation and repressive chromatin states and become aberrantly re-expressed in cancer through epigenetic deregulation [323–325]. Superimposed on this epigenetic permissiveness, tumor-specific TF networks, often driven by oncogenic signaling and master regulators like MYC, AP-1, STATs, or E2F family members, amplify and coordinate the expression of large fractions of the tumor transcriptome, thereby shaping both the composition and abundance of the TAA repertoire [326–328]. Remarkably, several TAAs targeted by autoantibodies are themselves transcriptional or chromatin-associated regulators, including p53, MYC, and topoisomerases, further linking oncogenic gene regulation to antigenic manifestation [316, 318].

In addition to transcriptional control, post-transcriptional regulation and antigen-processing constraints critically influence immune recognition. MicroRNAs modulate both TAA expression and epigenetic regulators, while frequent downregulation or mutation of MHC molecules and antigen-processing machinery reshapes the repertoire of presented TAA-derived peptides, enabling immune escape despite persistent antigen expression [329–331].

TAAs also serve as predictive biomarkers and therapeutic targets in immuno-oncology. Composite measures such as TAA burden correlate with improved clinical outcomes in patients receiving ICI treatment, supporting the concept that antigenic landscape, rather than single-antigen expression, influences immunotherapy responsiveness [332]. Therapeutically, TAAs are exploited by peptide vaccines and antibody-drug conjugates (ADCs). Long-term follow-up of multipeptide vaccination strategies has demonstrated improved overall survival in subsets of melanoma patients, while ADCs targeting surface-expressed TAAs, such as folate receptor alpha (FOLR1), provide targeted treatment options in selected cancers, albeit with modest response rates and treatment-related toxicities [333–337].

Overall, TAAs represent valuable diagnostic and therapeutic biomarkers whose clinical utility and limitations are dictated by transcriptional and epigenetic regulation, antigen-processing capacity, and tumor context. Integrating TAA measurements with transcriptional, epigenetic, and immune profiling will therefore be essential to enhance their diagnostic accuracy and therapeutic exploitation in precision oncology.

## Tumor microenvironment components as dynamic biomarkers and therapeutic targets

Cancer progression is driven by tumor-intrinsic genetic, epigenetic, and metabolic programs that actively reprogram the TME to promote immune evasion and therapeutic resistance. The TME is a dynamic ecosystem comprising of immune and stromal cells, endothelial components, and the ECM. Tumor-derived signals continuously shape the composition, functional state, and spatial organization of these components [293, 338]. Immune and stromal populations, including Tumor-infiltrating lymphocytes (TILs), CAFs, and TAMs, undergo transcriptional and epigenetic reprogramming in response to oncogenic signaling, cytokines, metabolic cues, and matrix remodeling (Fig. 2). Consequently, their abundance, differentiation state, and spatial distribution serve as cellular indicators of tumor-intrinsic regulatory programs, linking tumor biology to immune phenotype, therapeutic response and emerging strategies targeting the TME [339]. In this section, we discuss these key TME components as dynamic, integrative biomarkers relevant to cancer prognosis and immunotherapy.

### Tumor-infiltrating lymphocytes and CD8^+^ T cell states as predictive biomarkers

TILs represent a heterogeneous population of immune cells whose abundance, functional state, and spatial distribution reflect the balance between anti-tumor immunity and tumor-induced immune suppression. Higher TIL density is generally associated with improved clinical outcomes and enhanced responsiveness to immune checkpoint blockade (ICB) therapies across multiple cancer types, including melanoma, NSCLC, and gastrointestinal malignancies [340–343]. Among TIL subsets, CD8^+^ T cells have emerged as the most informative biomarkers due to their direct cytotoxic activity against tumor cells and their central role as ICI targets. Multiple studies have demonstrated that increased infiltration of CD8^+^ TILs correlates with favorable responses to PD-1/PD-L1 and CTLA-4 blockade in melanoma, head and neck squamous cell carcinoma, NSCLC, and gastric cancer [344–347]. Importantly, the spatial organization of CD8^+^ T cells within the TME strongly influences their predictive value. CD8^+^ TILs localized at the invasive tumor margin or within the stromal compartment, as well as close spatial proximity between PD-1^+^ CD8^+^ T cells and PD-L1^+^ tumor cells, have been associated with superior clinical responses to PD-1 blockade [348, 349]. These observations highlight that immune cell localization and tumor–immune cell interactions provide critical biomarker information beyond total cell counts.

Beyond abundance, functional heterogeneity and differentiation state of CD8^+^ T cells are key determinants of immunotherapy efficacy. CD8^+^ T cells differentiate into multiple functional subsets based on antigen stimulation, co-stimulatory signals, cytokine exposure, and transcriptional programs (Fig. 2). Cytotoxic Tc1 cells, progenitor-exhausted T cells, effector memory (Tem) and tissue-resident memory (Trm) subsets are consistently associated with improved responses to ICI, whereas terminally exhausted CD8^+^ T cells exhibit limited reinvigoration capacity [14, 350]. Combination ICI strategies targeting additional inhibitory receptors such as LAG-3, TIM-3, or TIGIT further underscore the importance of T cell state, as these therapies can reprogram exhausted CD8^+^ T cells toward more functional effector phenotypes [351].

Despite the strong biological rationale, the predictive value of CD8^+^ TILs remains inconsistent across studies. Discrepancies arise from patient-specific variables, tumor stage, cancer type, timing of tissue sampling, technical differences in detection methods, and failure to account for spatial context, functional exhaustion, or tertiary lymphoid structures within tumors [14, 352–357]. These limitations highlight that CD8^+^ TILs are not standalone biomarkers, but rather, cellular responses to upstream tumor-intrinsic transcriptional, epigenetic, and microenvironmental programs. Therefore, an integrative approach combining quantitative, spatial, and functional profiling of T cell populations is necessary to improve patient stratification and maximize the use of TIL-based biomarkers in immunotherapy.

### Cancer-associated fibroblasts as dynamic stromal biomarkers in cancer

CAFs are key stromal regulators of tumor progression and immune modulation within the TME. Single-cell and spatial omics technologies have revealed CAFs as a highly heterogeneous and plastic population derived from multiple cellular sources, including resident fibroblasts, endothelial and mesothelial cells, pericytes, adipocytes, monocytes, and bone marrow–derived mesenchymal stem cells [338]. Rather than forming discrete entities, CAFs occupy dynamic transcriptional states shaped by tumor-derived signals, cytokines, and epigenetic reprogramming, resulting in diverse effects on ECM remodeling, immune exclusion, and therapeutic response.

Although numerous CAF subtypes have been proposed (e.g., inflammatory, myofibroblastic, antigen-presenting, and tumor-like CAFs), accumulating evidence suggests that functional transcriptional programs are more informative than rigid subtype classification [338, 358]. CAF states enriched for ECM organization, TGF-β signaling, metabolic stress, or inflammatory pathways consistently associate with immune exclusion and poor prognosis across tumor types [359, 360]. Accordingly, CAF abundance and gene expression profiles are increasingly exploited as stromal biomarkers. For example, *CALD1* expression correlates with CAF infiltration and adverse outcomes in bladder cancer [361], whereas CAF-derived transcriptional signatures predict biochemical recurrence and metastasis-free survival in prostate cancer [362].

Importantly, CAFs are rarely used as standalone biomarkers. Instead, composite CAF-transcriptomic and CAF-epigenetic models are emerging as more robust predictors of prognosis and therapy response. In breast cancer, a CAF-related transcriptional signature derived from single-cell and bulk RNA sequencing stratifies survival and treatment response [363]. Similar multi-gene CAF signatures predict OS, immune infiltration, and sensitivity to chemotherapy or immunotherapy in colorectal, lung, and other cancers [364–367]. Spatial transcriptomic studies further demonstrate that combining pan-CAF markers (e.g., α-SMA, FAP, PDGFRα/β) with subtype-specific transcriptional programs yields independent prognostic information in breast and pancreatic cancer [368–371].

Epigenetic profiling adds an additional, clinically relevant layer. CAFs exhibit tumor-driven DNA methylation changes correlated with gene expression and patient outcome. CAF-specific CpG sites, including EDARADD and HDAC4 loci, have been linked to survival across multiple cancer types [372]. Integrating CAF abundance with differentially methylated and expressed genes produced a composite risk score in lower-grade glioma that outperformed single-layer biomarkers for prognosis and prediction of ICI and chemotherapy response [373]. Similar CAF-epigenetic combinations improve risk stratification in prostate cancer, indicating that epigenetic “memory” within CAFs captures stable resistance states not evident from transcriptional data alone [374].

Functionally, CAF plasticity underlies both tumor-promoting and tumor-restraining activities, with anti-tumor effects more prominent in early disease stages and immunosuppressive phenotypes dominating during progression [338, 375]. This plasticity likely explains the limited success of CAF-targeting strategies in clinical trials, where non-selective CAF depletion has, in some cases, accelerated tumor growth and worsened outcomes [338, 376]. Collectively, these observations indicate that CAFs function as integrative stromal biomarkers that are best interpreted through multi-parameter models combining abundance, transcriptional state, epigenetic features, and spatial context rather than single markers alone.

### Tumor-associated macrophage states as biomarkers of tumor-intrinsic immune reprogramming

TAMs are clinically relevant biomarkers due to their high abundance within tumors, reaching up to 50% of the tumor mass in some cancers, as well as their functional plasticity and spatial organization within the TME. Tumor-derived signals recruit circulating monocytes that differentiate into TAMs, whose density and phenotype are strongly associated with patient outcomes. Immunohistochemical quantification of total macrophage infiltration using CD68 generally correlates with poor prognosis across multiple malignancies, including melanoma, hepatocellular carcinoma, glioblastoma, and cancers of the lung, pancreas, stomach, breast, ovary, and prostate [377]. Colorectal cancer is a notable exception in which increased TAM density, particularly when accompanied by robust T cell infiltration, is associated with a better prognosis. This highlights the context-dependent roles of macrophages and their cooperation with adaptive immunity [378].

In addition to total abundance, TAM phenotypes can be refined using additional markers such as TREM2, CD163, CD206, FOLR2, and SPP1, which better capture functional states linked to prognosis and therapy response [377, 379]. For example, expression of the glycolytic regulator PFKFB3 in TAMs correlates with M2-like polarization, increased macrophage infiltration, tumor growth, and reduced overall survival in colorectal cancer [379]. While TAMs were historically classified along an M1/M2 axis, single-cell and spatial transcriptomic studies now reveal multiple transcriptionally distinct TAM states that are not adequately described by this dichotomy, underscoring the need for state-based rather than binary classification [380, 381].

Importantly, TAM recruitment and polarization are not autonomous immune phenomena but are actively imposed by tumor-intrinsic transcriptional and metabolic programs. Cancer cells secrete chemokines and growth factors such as CCL2, CCL5, CSF1, VEGF, IL-10, and TGF-β that recruit monocytes and stabilize immunosuppressive macrophage programs [382–384]. These cues are reinforced by tumor metabolism and hypoxia, where lactate, adenosine, and nutrient competition activate HIF-dependent transcriptional circuits that favor M2-like, tissue-remodeling states [385–388]. Tumor-derived extracellular vesicles carrying non-coding RNAs further rewire macrophage gene expression, while extracellular matrix remodeling and fibrosis impose additional TGF-β-dependent constraints that suppress effector T cell activity [383, 389, 390]. These integrated tumor-driven programs convert macrophages into stable, tumor-educated TAM states that promote angiogenesis, invasion, immune evasion, and resistance to therapy [384, 391].

The spatial distribution of TAMs within the TME further refines their biomarker value. TAM signatures localized to the tumor core are often associate with poor outcomes, whereas enrichment within tertiary lymphoid structures correlates with improved survival, reflecting localized immune activation [377, 392]. Similarly, FOLR2^+^ perivascular TAMs correlate with enhanced CD8^+^ T cell infiltration and favorable prognosis in breast cancer, whereas SPP1^+^ TAMs cooperate with FAP^+^ CAFs to drive desmoplasia, T cell exclusion, and resistance to immune checkpoint blockade across several tumor types [393–395].

Beyond tissue-based analyses, circulating TAM-derived factors such as soluble CD163 and TREM2 provide minimally invasive readouts of tumor-induced immunosuppression and have shown promise as biomarkers of immunotherapy response [396]. From a therapeutic standpoint, strategies that exploit macrophage effector functions, particularly the blockade of “do not eat me” signals like CD47, demonstrate that TAMs can be pharmacologically redirected, although durable clinical benefit remains limited [397–399].

In summary, TAMs act as dynamic, spatially resolved biomarkers that reflect tumor-intrinsic transcriptional, metabolic, and stromal programs rather than isolated immune processes. Their abundance, phenotype, and localization capture the integrated state of tumor-driven immune reprogramming, supporting their use as contextual biomarkers best interpreted alongside transcriptional, epigenetic, and spatial immune profiling approaches [377].

## Artificial intelligence-driven integration of multi-omics data in biomarker discovery and clinical oncology

The biological complexity of cancer increasingly challenges traditional biomarker paradigms based on single molecular features. Genetic alterations, epigenetic states, transcriptional programs, immune composition, spatial organization, and clinical variables interact in nonlinear ways that cannot be fully captured by isolated biomarkers. Consequently, there is growing recognition that robust prediction of cancer progression and therapeutic response requires integrative, multidimensional biomarker frameworks [400, 401]. Artificial intelligence (AI) and machine learning (ML) approaches have emerged as powerful tools to address this challenge by enabling the systematic integration and interpretation of high-dimensional, multi-omics data [402, 403], thereby generating composite biomarkers that more accurately reflect tumor biology and support improved clinical decision-making (Fig. 2).

### AI-driven biomarker discovery and clinical applications

AI-based methods are already demonstrating value in several areas of biomarker research and translational oncology. In digital pathology, deep learning models applied to routine hematoxylin and eosin (H&E)-stained slides can infer molecular alterations, immune phenotypes, and prognostic risk without additional assays. Such approaches have been shown to predict TMB, MSI, immune infiltration patterns, and response to ICI across multiple cancer types [352, 404–407]. Importantly, these models function as decision-support tools, augmenting rather than replacing pathological assessment [403, 408].

Importantly, AI-based digital pathology is increasingly being used to quantify and spatially interpret the immune and stromal biomarkers discussed earlier in this review. Deep learning models applied to PD-L1 immunohistochemistry whole-slide images can perform automated tumor proportion score (TPS) or combined positive score (CPS) assessments that are highly concordant with those of expert pathologists. This reduces interobserver variability and improves reproducibility in clinical workflows [409–411]. Likewise, AI-powered image analysis can automatically detect and quantify TILs in routine histology or immunostained slides. Beyond simple cell counts, spatial analysis of TIL distribution allows tumors to be classified as inflamed, immune-excluded, or immune-desert, which strongly correlates with response to immune checkpoint inhibitors and clinical outcome [412–416]. Emerging approaches extend these analyses to stromal components. AI-based quantification of tumor-stroma architecture and fibroblast-rich regions has begun to capture cancer-associated fibroblast heterogeneity and its association with immune exclusion and recurrence risk [417, 418]. Together, these developments illustrate how AI-driven spatial analysis can translate tumor-intrinsic regulatory programs into measurable microenvironmental biomarker patterns. These spatially resolved analyses therefore provide a computational bridge between tumor-intrinsic transcriptional programs and the cellular and spatial biomarkers described in earlier sections of this review, including PD-L1 expression, TIL states, CAF heterogeneity, and macrophage polarization.

Beyond molecular inference, AI-based analysis of routine histopathology has demonstrated prognostic value across multiple tumor types. Deep learning models trained on standard H&E slides can stratify colorectal, prostate, and renal cancer patients into clinically meaningful risk groups, predicting recurrence, tumor grade, and survival with performance comparable to expert pathologists and, in some settings, established genomic assays [419–421]. These studies illustrate how AI can extract biomarker-relevant information from existing diagnostic material, supporting risk stratification without additional tissue sampling or molecular testing.

Similarly, radiomics provides a non-invasive route to capture tumor heterogeneity and treatment response by combining quantitative features from CT, PET, or MRI with machine-learning models. When integrated with genomic and clinical data, radiomic signatures improve prognostic assessment and therapy selection in lung, brain, and gastrointestinal cancers [422–424]. More broadly, AI-driven risk prediction extends beyond tumor-specific biomarkers: ML models applied to electronic health record data already support clinical decision-making in emergency and chronic care settings, outperforming traditional scoring systems for outcomes such as hospitalization and mortality [425, 426]. Together, these applications highlight how AI-based biomarkers function as integrative decision-support tools embedded within clinical workflows rather than isolated predictive readouts.

Beyond imaging and clinical data, AI enables the integration of diverse molecular layers, including genomics, transcriptomics, epigenomics, proteomics, and single-cell or spatial data, into composite biomarker models. Multi-omics machine-learning frameworks have been shown to predict survival, drug sensitivity, and immunotherapy response more accurately than any single data modality alone [401, 427, 428]. In breast and ovarian cancer, such integrative models stratify patients into molecular risk groups associated with clinical outcome while also nominating candidate therapeutic strategies for high-risk subgroups [429, 430].

Importantly, these approaches do not replace individual biomarkers but instead position them within broader integrative frameworks that reflect underlying biological programs. As such, they are particularly well-suited to capturing the transcriptional and epigenetic regulation, tumor-immune interactions, and tumor microenvironmental states that have been highlighted throughout this review.

### Methodological considerations and interpretability

While AI-driven biomarker models offer substantial promise, their clinical translation requires careful methodological consideration. Most current studies are retrospective and rely on curated datasets that may not fully capture real-world biological and clinical heterogeneity. Therefore, generalizability across institutions, analytical platforms, and patient populations remains a major challenge [431]. In addition, clinical adoption of model depends not only on predictive performance but also on the ability of clinicians to understand and trust their outputs.

Several methodological strategies are emerging to address these limitations. Causal machine-learning frameworks are increasingly being explored to estimate individualized treatment effects and distinguish predictive biomarkers from purely prognostic correlates. By explicitly modeling treatment-outcome relationships rather than relying on correlations alone, these approaches aim to improve the clinical reliability of AI-derived biomarkers [432]. Early applications include decision-support systems that predict therapy-specific toxicities, complications, or benefit-risk profiles, thereby assisting clinicians in tailoring treatment intensity and selection [433].

At the same time, there is a growing emphasis on explainable and interpretable AI. Instead of treating models as opaque predictors, explainable AI approaches aim to identify biologically meaningful features that drive predictions, such as TF programs, immune cell states, stromal signatures, and spatial patterns [434–437]. This is particularly relevant in immuno-oncology, where mechanistic insight is essential for biomarker validation, regulatory acceptance, and clinical decision-making. Causal modeling and explainable AI frameworks complement each other, providing strategies to enhance the robustness, transparency, and translational relevance of AI-based biomarker models.

### Emerging perspectives: quantum computing and future integration

Looking ahead, emerging computational paradigms may further expand the scope of biomarker discovery. Quantum computing, although still in an early developmental phase, offers theoretical advantages for solving complex optimization and pattern-recognition problems that scale poorly on classical hardware. In oncology and systems biology, quantum and quantum-inspired approaches have been proposed for exploring large combinatorial biomarker spaces, optimizing feature selection in multi-omics datasets, and accelerating molecular simulations [438–440]. Early proof-of-concept studies have demonstrated this potential. Hybrid quantum-classical workflows have also been applied to drug discovery and biomarker modeling, including quantum-enhanced generative models that identify candidate KRAS inhibitors and quantum machine-learning frameworks that improve drug sensitivity prediction using proteomic data [441, 442].

At present, quantum computing is not ready for routine clinical biomarker deployment. Hardware limitations, noise, and the lack of large-scale validation remain major barriers. Nevertheless, hybrid models that integrate classical AI with quantum-inspired optimization algorithms are actively being explored and may, in the long term, complement multi-omics pipelines where classical approaches encounter computational limits [443, 444]. Therefore, these developments should be viewed as future-enabling technologies rather than immediate clinical solutions.

## Concluding perspective

Cancer biomarkers are increasingly recognized not as static molecular features, but as dynamic manifestations of tumor-intrinsic transcriptional and epigenetic programs that actively shape immune interactions, stromal remodeling, and therapeutic vulnerability. This review highlights a unifying principle across genetic, epigenetic, immune, and microenvironmental layers: clinically relevant biomarkers emerge from coordinated gene regulatory networks rather than isolated alterations.

By integrating tumor-intrinsic regulators with cellular and spatial features of the TME biomarkers gain explanatory power, contextual robustness, and improved predictive value. This perspective helps reconcile inconsistencies observed across single-marker studies and explains why biomarker performance is often cancer-type – and context-dependent.

Advances in multi-omics profiling, spatial technologies, and AI-driven integration now make it feasible to implement this regulatory framework, enabling composite biomarkers that better capture tumor plasticity and immune dynamics. Although methodological and translational challenges remain, particularly regarding standardization and interpretability, these approaches mark a transition toward systems-level biomarker strategies in precision oncology.

Ultimately, anchoring biomarker discovery in transcriptional and epigenetic regulation provides a coherent conceptual foundation for future diagnostics and therapeutic stratification, bridging mechanistic tumor biology with clinically actionable decision-making.

## Data Availability

No datasets were generated or analysed during the current study.

## Abbreviations

AI: Artificial intelligence
AML: Acute myeloid leukemia
APM: Antigen-processing machinery
APC: Antigen-presenting cell
CAF: Cancer-associated fibroblast
CAR-T: Chimeric antigen receptor T cell
CD: Cluster of differentiation
CSC: Cancer stem cell
CTL: Cytotoxic T lymphocyte
CTC: Circulating tumor cell
CTA: Cancer/testis antigen
DC: Dendritic cell
dMMR: Deficient mismatch repair
DNA: Deoxyribonucleic acid
ECM: Extracellular matrix
EGFR: Epidermal growth factor receptor
EMT: Epithelial-mesenchymal transition
EMP: Epithelial-mesenchymal plasticity
E2F: E2F transcription factor
FFPE: Formalin-fixed paraffin-embedded
GC: Gastric cancer
H&E: Hematoxylin and eosin
HIF: Hypoxia-inducible factor
HLA: Human leukocyte antigen
HNSCC: Head and neck squamous cell carcinoma
ICI: Immune checkpoint inhibtion
IFN: Interferon
IHC: Immunohistochemistry
IL: Interleukin
MDSC: Myeloid-derived suppressor cell
MHC: Major histocompatibility complex
miRNA: MicroRNA
ML: Machine learning
MMR: Mismatch repair
MRI: Magnetic resonance imaging
MSI: Microsatellite instability
MSI-H: Microsatellite instability-high
ncRNA: Non-coding RNA
NGS: Next-generation sequencing
NK cell: Natural killer cell
NSCLC: Non-small cell lung cancer
OS: Overall survival
PD-1: Programmed cell death protein 1
PD-L1: Programmed death-ligand 1
PET: Positron emission tomography
PFS: Progression-free survival
RNA: Ribonucleic acid
scRNA-seq: Single-cell RNA sequencing
SNP: Single nucleotide polymorphism
TAAs: Tumor-associated antigens
TAM: Tumor-associated macrophage
TCR: T cell receptor
TF: Transcription factor
TIL: Tumor-infiltrating lymphocyte
TMB: Tumor mutational burden
TME: Tumor microenvironment
TLS: Tertiary lymphoid structure
Trm: Tissue-resident memory T cell
